# The Methodological Quality of Case Series Published Early vs. Late in the Course of a Pandemic: A Meta-Epidemiologic Study

**DOI:** 10.1055/s-0045-1806762

**Published:** 2025-04-02

**Authors:** Yahya Alsawaf, Mohammed Firwana, Tarek Nayfeh, Mohamed O. Seisa, Reem A. Alsibai, Alzhraa S. Abbas, Elizabeth H. Lees, Ye Zhu, Michael E. Wolf, Greg Vanichkachorn, Moustafa Hegazi, Larry J. Prokop, M. Hassan Murad, Samer Saadi

**Affiliations:** 1 Evidence-based Practice Center, Kern Center for the Science of Healthcare Delivery, Mayo Clinic, Rochester, Minnesota, United States; 2 Division of Public Health, Infectious Diseases and Occupational Medicine, Mayo Clinic, Rochester, Minnesota, United States; 3 Mayo Clinic Library System Evidence-based Practice Center, Kern Center for the Science of Healthcare Delivery, Mayo Clinic, Rochester, Minnesota, United States

**Keywords:** methodological quality, risk of bias, meta-epidemiological research, case series, case reports

## Abstract

**Introduction** Case reports and series are critical to guide initial decision-making in a pandemic, but may have lower rigor because of the need to publish them quickly. This meta-epidemiologic study compares the methodological quality of case series that described the acute coronavirus disease 2019 (COVID-19) pandemic in 2020 versus those that described long-haul cases.

**Methods** We conducted a systematic review in multiple databases for long-haul case series and reports. We identified early cases of acute COVID-19 synthesized in published systematic reviews. We evaluated the methodological quality by pairs of independent reviewers using a tool dedicated for appraising case series.

**Results** We included 239 original case series (81 published in the first year of the pandemic and 158 published later describing long-haul COVID). The methodological quality of both groups of case series was very good (80–100% of series satisfying quality items) except for two items, the selection approach of cases included in the series and ruling out other causes that can explain the main finding described in the series. The appraisal tool demonstrated high agreement and reliability between reviewers.

**Conclusion** The methodological quality of modern case series is high, except for two quality items that represent an area for potential for improvement for authors of case series describing future pandemics.

## Introduction

Case reports and series represent a critical study design in medicine and epidemiology. For example, in 1910 Dr. James Herrick published about a patient with abnormal red blood cells, which defined a new condition called sickle cell disease. 1 In an epidemic or a pandemic, these series are even more crucial and are often published quickly. A case series in 1981 described five young men in Los Angeles with rare *Pneumocystis jirovecii* pneumonia heralded the human immunodeficiency virus/acquired immunodeficiency syndrome epidemic. 2 Another case series of 41 patients hospitalized with pneumonia in Wuhan, China, established the coronavirus disease 2019 (COVID-19) pandemic. 3 Case reports and series can clarify the presentation of patients in a pandemic and describe their treatments, outcomes, and clinical course, which facilitate the design of subsequent rigorous trials. Therefore, improving the methodological quality of these early case reports and series is critical.

Early case series in the course of a pandemic may be rushed or authored by clinicians or researchers with less research expertise, or may have an accelerated publication course. As a result, their methodological quality may be lower than that of case series published later, such as those describing a chronic or subsequent complication of the same epidemic. To test this hypothesis, and with the aim of improving the methodological quality of case series published in pandemics, we compared the quality of case series that were published early in the course of the COVID-19 pandemic to case series that described its chronic sequela, long-haul COVID.

## Methods

This methodological study adheres to the reporting guidelines of meta-epidemiology research. 4 The study follows a priori established inclusion and exclusion criteria.

### Data Sources and Search Strategies

For case reports and series of long-haul COVID, we searched MEDLINE(R) and Epub Ahead of Print, In-Process and Other Non-Indexed Citations, and Daily, EMBASE, Cochrane Central Register of Controlled Trials, Cochrane Database of Systematic Reviews, PsycINFO, and Scopus from the beginning of 2019 to April 21, 2022. Controlled vocabulary supplemented with keywords was used to search for physical, cognitive, and occupational impairment in adults infected with COVID-19. The search strategy was designed and conducted by a medical reference librarian with input from the study investigators. We excluded case reports and series about children or adolescents (age < 18) and non-English language publications. The detailed strategy is provided in the Supplementary Appendix (online only). For case reports and series of the original COVID-19 pandemic (the first year of the pandemic), which were already synthesized and appraised by numerous systematic reviews, we identified these publications through existing systematic reviews. We adopted a back-citation approach by identifying in Scopus the systematic reviews that have referenced the methodological quality assessment tool designed for case series and case reports (Murad tool, BMJ Evidence-Based Medicine). 5 We applied filters to include systematic reviews published in 2020, written in English, and with the term “COVID” in their title. We eventually included 6 eligible systematic reviews 6 7 8 9 10 11 from which we extracted 81 case reports/series.

### Methodological Quality Assessment

The methodological quality assessment tool designed for case series and case reports has been used in over a thousand systematic reviews 5 and evaluates four domains: selection, ascertainment, causality, and quality of reporting. These four domains have eight signaling questions (subdomains), from which two do not apply to the current topic (challenge/rechallenge and dose–response gradient), leaving the following six subdomains that were assessed in this study:

- selection methods of the patients,
- ascertainment of the exposure,
- ascertainment of the outcome,
- causality (i.e., ruling out other causes that may explain the main observation or manifestation described in the case series),
- causality (sufficient follow-up time), and
- the quality of reporting (is the case(s) described with sufficient details to allow other investigators to replicate the research or to allow practitioners make inferences related to their own practice?).

Two independent reviewers used the tool to appraise the long-haul COVID-19 series. Disagreements were resolved by discussion between the two reviewers. The methodological quality of the early pandemic cases was extracted from the existing systematic reviews. These reviews did not report quantitative measures of agreement and used a consensus process. To verify the quality assessment extracted from the published systematic reviews, we verified a 10% sample that we reappraised and was consistent with the published appraisals.

### Analysis

We compared the methodological quality of the series published in the first year of the pandemic describing acute COVID-19 manifestations to that of series published later in the pandemic describing long-haul COVID syndrome. We used Fisher's exact test to compare the proportions of case series that satisfied each of the six individual methodological quality items. Two-tailed values were considered statistically significant if *p* < 0.05. Analysis was done in R (version 4.1.3 [2022–03–10], R Core Team [2021]; R: A language and environment for statistical computing; R Foundation for Statistical Computing, Vienna, Austria).

## Results

After screening 11,702 citations, we included 239 original case series (81 published in the first year of the pandemic and 158 published later describing long-haul COVID). These studies are included in the Supplementary Appendix ( Supplementary Table S1 , available online only). The process of study selection is depicted in Supplementary Fig. S1 in the Supplementary Appendix (available online only).

### Overall Methodological Quality

The observed agreement among the reviewers about the six quality items was excellent (83, 100, 100, 84, 100, and 99%, respectively). In general, the methodological quality of both groups of case series was very good. The highest domains were ascertainment of the exposure (238/239, 99.6%), ascertainment of the outcome (231/239, 96.7%), sufficient follow-up time (232/239, 97.1%), and quality of reporting (218/239, 91.2%). The lowest domains were ruling out other causes that can explain the finding/phenomenon described in the case series (87/191, 45.6%), followed by the selection of cases included in the series (180/239; 75.3%).

### Comparison between the Two Groups

**Fig. 1:**
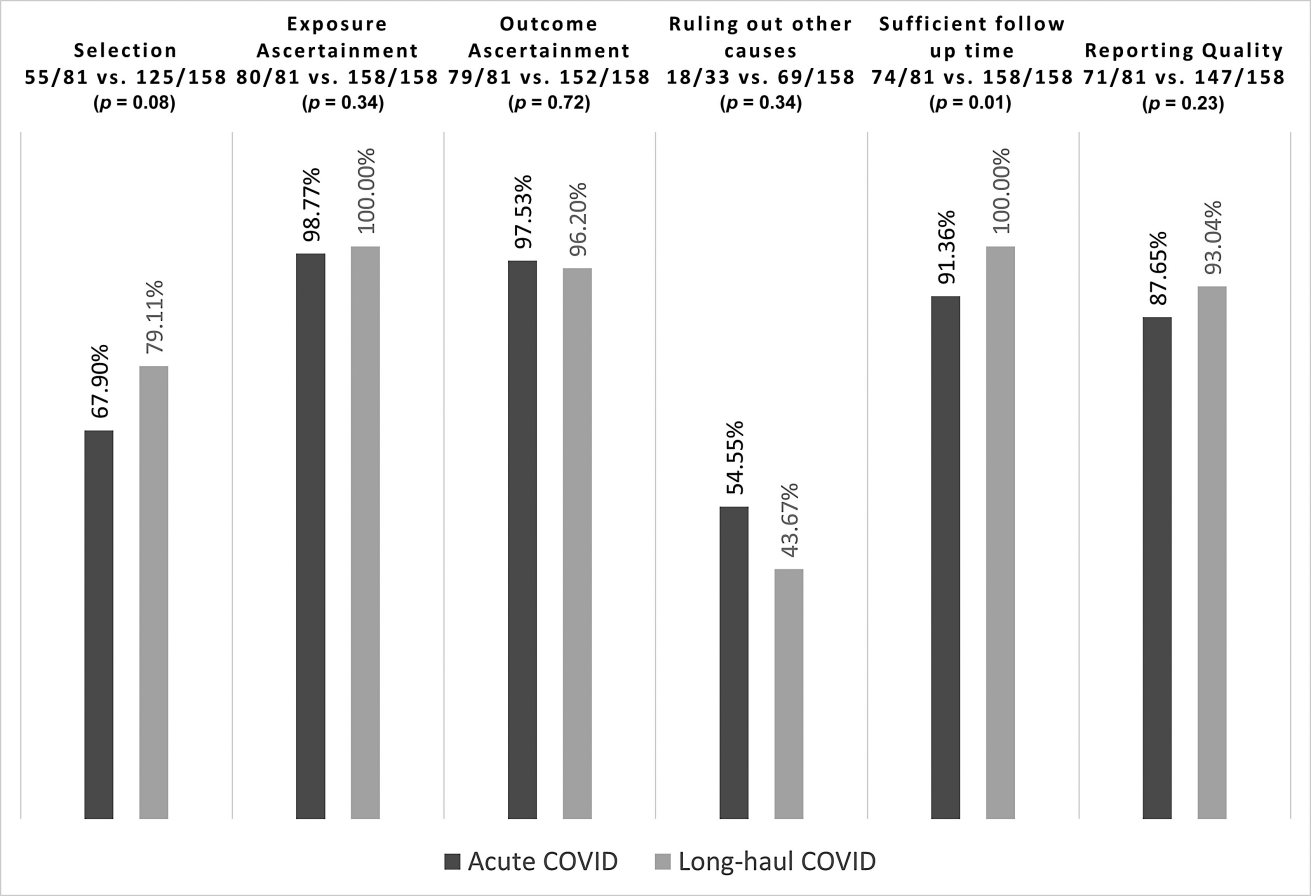
The proportions of case series that satisfied the respective methodological quality domains/subdomains. *p* -Values are derived from Fisher's test.

In Fig. 1 , we depict the proportions of case series that satisfied the six quality items of the tool along with *p* -values for the difference between the two groups. The methodological quality of long-haul series was better than that of acute series for all domains except ruling out other causes (43.7% vs. 54.5%) and exposure ascertainment (near perfect in both groups). The difference was statistically significantly only for a single quality item, the causality item about sufficient follow-up time (100% vs. 91.4%, *p* = 0.01).

## Discussion

Case series and reports are considered a study design that provides weak inferences; however, they are critical publications particularly in pandemics. Since they are published promptly at the beginning of a pandemic, we hypothesized that their quality may not be adequate. With the aim of improving the quality of case reports and series during a pandemic, we conducted this brief analysis. We assessed the methodological quality of case series published in the first year of the COVID-19 pandemic and compared them to those published later describing long-haul COVID-19. There was a statistically significant difference in a single quality item (the causality item about sufficient follow-up time). However, long-haul cases had a minimum of 2-week period between infection and persistence of symptoms as an inclusion criterion, and thus satisfied this criterion by definition. In addition, the difference in the proportions of series satisfying each item was small. Therefore, contrary to our hypothesis, the quality of case series in both periods was overall excellent. The appraisal tool demonstrated high agreement and reliability between reviewers.

### Implications

The current analysis demonstrated a potential for improving the methodological quality of case series in two areas.

The first is about the selection of cases included in the series. For example, one of the included case series reported on 118 patients who presented for outpatient follow-up with symptoms that persisted for at least 2 weeks after being diagnosed with severe acute respiratory syndrome coronavirus 2 (SARS-CoV-2) infection. 12 This published report does not explicitly mention whether these 118 patients were all the patients with persistent symptoms in the health systems of the investigators, whether they were selected based on certain criteria out of a larger sample, whether some were excluded for loss to follow-up or simply for not showing up after the acute infection, and does not report a denominator (the total number of patients diagnosed with SARS-CoV-2 infection). Knowing such a denominator could have allowed the estimation of a prevalence of long-haul syndrome. In future pandemics we strongly encourage authors of case series to report such information, to which they likely have access, to allow better inferences.

The second area of possible improvement that we noted related to the quality item about ruling out other causes that can explain the finding/phenomenon described in the case series. For example, a case series reported on 39 patients who reported residual symptoms 48 weeks after discharge from a COVID-19-related hospitalization, particularly, fatigue, dyspnea, and difficulty in concentration. 13 Although the questionnaires used to interview the patients asked whether some of the symptoms were “unchanged compared to before COVID-19,” this was not done for all symptoms. Furthermore, the publication did not mention any attempts to determine whether these symptoms had other plausible reasons, such as preexisting morbidities or other etiologies, or whether the patients had any diagnostic testing for these symptoms. Thus, the attribution of these symptoms to SARS-CoV-2 infection is challenging. Conversely, a publication that described the first 100 patients with long-haul syndrome presenting to an academic center reported on the diagnostic tests and consultations that were done to exclude other causes for the symptoms that persisted after SARS-CoV-2 infection. 14 We encourage future authors of case series to address this causality item of the tool by attempting to exclude other potential causes of the phenomenon described in the case series.

## Strengths and Limitations

The current study used a systematic approach to identify and appraise eligible publications. The quality assessment tool has been used in over a thousand reviews and demonstrated adequate reliability. The findings about the methodological quality of case series and recommendations to improve them can be extrapolated to case series describing future pandemics.

In terms of limitations, the risk of bias and quality of reporting are separate constructs. However, in case reports and case series, this separation becomes less clear because these studies often do not provide estimates of effect that can be biased in a statistical or epidemiological sense. Therefore, the tool does include an item about reporting ( *Is the case(s) described with sufficient details to allow other investigators to replicate the research or to allow practitioners make inferences related to their own practice?* ). 5 We also acknowledge a terminology challenge in which some case series can also be described as an uncontrolled single-arm cohort study. For example, two included case series that reported on patients with persistent symptoms after SARS-CoV-2 infection were labeled by their authors as a cohort study, 13 14 which is appropriate because of some longitudinal and prospective elements in these studies. This terminology issue has been long recognized in the methodological literature with various proposed solutions, and is debated elsewhere. 5 15 16 17 Lastly, we identified case series of acute COVID-19 from existing systematic reviews because these series have already been extensively synthesized and we did not see the need to reconduct these systematic reviews. Limitations and omissions of these systematic reviews can extend to this meta-epidemiologic study.

## Conclusion

Future pandemics will be first described via case reports and series. Producing these publications with high rigor and methodological quality is critical for patient care early in the course of a pandemic and can guide the design of subsequent large randomized trials that can provide more certain and reliable evidence to guide decision-making.

## What is New?

- The methodological quality of case series about acute COVID-19 published early in the course of the pandemic was adequate.
- The methodological quality of case series about long-haul COVID-19 published later in the course of the pandemic was adequate.
- This study identifies two areas for possible improvement in future case series about a pandemic.
- The quality assessment tool for case series demonstrated high agreement and reliability between reviewers.
