# Supplementary material for: The Methodological Quality of Case Series Published Early vs. Late in the Course of a Pandemic: A Meta-Epidemiologic Study
Source: Avicenna J Med. 2025 Apr 2;15(1):29–33. doi: 10.1055/s-0045-1806762 (PMC12088789; doi:10.1055/s-0045-1806762)
Supplement: Supplementary file 1 — Supplementary Material [file 10-1055-s-0045-1806762-s240088.pdf]

Supplementary Appendix

Search Strategy

Ovid

Database(s): APA PsycInfo 1806 to April Week 3 2022, EBM Reviews - Cochrane Central Register of Controlled Trials March 2022, EBM Reviews - Cochrane Database of Systematic Reviews 2005 to April 20, 2022, Embase 1974 to 2022 April 21, Ovid MEDLINE(R) and Epub Ahead of Print, In-Process, In-Data-Review & Other Non-Indexed Citations, Daily and Versions 1946 to April 21, 2022

Search Strategy:

#	Searches	Results
1	exp COVID-19/	375091
2	(((“Corona virinae” or “corona virus” or Coronavirinae or coronavirus or COVID or nCoV or hCoV) adj4 (“19” or “2019” or novel or new or nouveau or nuevo)) or (pneumon* adj3 Wuhan) or ((“Corona virinae” or “corona virus” or Coronavirinae or coronavirus* or COVID or nCoV or hCoV) and (wuhan or china or chinese or hubei)) or “2019-novel Cov” or “2019-ncov” or “COVID-19” or “COVID2019” or “COVID 2019” or “Corona virinae19” or “Corona virinae2019” or “corona virus19” or “coronavirus-19” or “corona virus2019” or Coronavirinae19 or Coronavirinae2019 or coronavirus19 or coronavirus2019 or “coronavirus-2019” or COVID19 or COVID2019 or nCOV19 or ncov-19 or nCOV2019 or “SARS Corona virus 2” or “SARS-coronavirus2” or “SARS-coronavirus-2” or “SARS-COV-2” or “SARS-COV2” or “Severe Acute Respiratory Syndrome Corona virus 2” or “Severe Acute Respiratory Syndrome Coronavirus 2” or sarscov*).ti,ab,hw,kw,mp. or ((coronavir* or COVID*) adj5 (pandemic* or outbreak or shutdown or “shut down*” or “shut-down*” or quarantin* or (lock* adj down) or “lock-down*” or lockdown* or “stay at home” or “stay-at-home” or “shelter-in-place”)).ti. or (Severe Acute Respiratory Syndrome Coronavirus 2 or COVID-19 or COVID-19 drug treatment or COVID-19 serotherapy or COVID-19 diagnostic testing or COVID-19 vaccine or spike glycoprotein, COVID-19 virus).os,ps,rs,ox,px,rx,nm.	552170
3	1 or 2	552330
4	Cognitive Dysfunction/	191833
5	cognitive defect/ or mild cognitive impairment/	254894
6	cognitive impairment/	261253
7	“activities of daily living”/ or functional status/	224344
8	fatigue/ or exp mental fatigue/	285746
9	Muscle Weakness/	61871
10	Occupational Health/	84588
11	Occupational Therapy/	46009
12	((safety adj4 sensitive) or “activities of daily living” or “cognitive decline*” or “cognitive deficit*” or “cognitive disabilit*” or “cognitive dysfunction*” or “cognitive impairment*” or “daily life activit*” or fatigue or “functional capacity” or “functional status” or “job duties” or “job duty” or “mental deterioration*” or “Muscle Weakness” or “neurocognitive disorder*” or “occupational duties” or “occupational duty” or “occupational health*” or “occupational therap*” or “physical impairment*” or “work duties” or “work duty”).ti,ab,kf.	1100779
13	or/4–12	1549356
14	3 and 13	20240
15	limit 14 to english language [Limit not valid in CDSR; records were retained]	19608
16	limit 14 to no language specified [Limit not valid in APA PsycInfo,CDSR; records were retained]	530
17	15 or 16	19748
18	limit 17 to yr = ”2019 -Current”	19715
19	(newborn* or neonat* or infant* or toddler* or child* or adolescent* or pediatric* or pediatric* or girl or girls or boy or boys or teen or teens or teenager* or preschooler* or “pre-schooler*” or preteen or preteens or “pre-teen” or “pre-teens” or youth or youths).ti,ab,hw,kf.	10665153
20	(adult or adulthood or adults or centenarian* or elderly or geriatric* or “middle age” or “middle aged” or nonagenarian* or octogenarian* or “old adult*” or “old people” or “old person*” or “older adult*” or “older people” or “older person*” or septuagenarian* or Sextenarian* or “very old”).ti,ab,hw,kf.	19380038

 © 2025. The Author(s).

(*Continued*)

#	Searches	Results
21	19 not 20	6423599
22	18 not 21	18882
23	limit 22 to (dissertation abstract or conference abstract or editorial or erratum or note or addresses or autobiography or bibliography or biography or blogs or comment or dictionary or directory or interactive tutorial or interview or lectures or legal cases or legislation or news or newspaper article or overall or patient education handout or periodical index or portraits or published erratum or video-audio media or webcasts) [Limit not valid in APA PsycInfo,CCTR,CDSR,Embase,Ovid MEDLINE(R),Ovid MEDLINE(R) Daily Update,Ovid MEDLINE(R) PubMed not MEDLINE,Ovid MEDLINE(R) In-Process,Ovid MEDLINE(R) Publisher; records were retained]	2992
24	22 not 23	15890
25	limit 24 to yr = ”2022 -Current”	2894
26	remove duplicates from 25	1889
27	limit 24 to yr = ”2021”	8569
28	“covid 19.”ti.	353044
29	27 and 28	5981
30	remove duplicates from 29	4327
31	27 not 28	2588
32	remove duplicates from 31	1942
33	limit 24 to yr = ”2019 -2020”	4427
34	remove duplicates from 33	3399
35	26 or 30 or 32 or 34	11557

Scopus

1. TITLE-ABS-KEY(((“Corona virinae” or “corona virus” or Coronavirinae or coronavirus or COVID or nCoV or hCoV) W/4 (“19” or “2019” or novel or new or nouveau or nuevo)) OR ((“Corona virinae” or “corona virus” or Coronavirinae or coronavirus* or COVID or nCoV or hCoV) and (wuhan or china or chinese or hubei)) OR (pneumon* W/3 Wuhan) OR “2019-ncov” OR “2019-novel Cov” OR “Corona virinae19” OR “Corona virinae2019” OR “corona virus19” OR “corona virus2019” OR Coronavirinae19 OR Coronavirinae2019 OR coronavirus19 OR “coronavirus-19” OR coronavirus2019 OR “coronavirus-2019” OR “COVID 2019” OR COVID19 OR “COVID-19” OR COVID2019 OR “ncov-19” OR nCOV19 OR nCOV2019 OR “SARS Corona virus 2” OR “SARS-coronavirus2” OR “SARS-coronavirus-2” OR sarscov* OR “SARS-COV2” OR “SARS-COV-2” OR “Severe Acute Respiratory Syndrome Corona virus 2” OR “Severe Acute Respiratory Syndrome Coronavirus 2”)
2. TITLE(((coronavir* or COVID*) W/5 (pandemic* or outbreak or shutdown or “shut down*” or “shut-down*” or quarantin* or (lock* W/1 down) or “lock-down*” or lockdown* or “stay at home” or “stay-at-home” or “shelter-in-place”)))
3. 1 or 2
4. TITLE-ABS-KEY((safety W/4 sensitive) OR “activities of daily living” OR “cognitive decline*” OR “cognitive deficit*” OR “cognitive disabilit*” OR “cognitive dysfunction*” OR “cognitive impairment*” OR “daily life activit*” OR fatigue OR “functional capacity” OR “functional status” OR “job duties” OR “job duty” OR “mental deterioration*” OR “Muscle Weakness” OR “neurocognitive disorder*” OR “occupational duties” OR “occupational duty” OR “occupational health*” OR “occupational therap*” OR “physical impairment*” OR “work duties” OR “work duty”)
5. PUBYEAR AFT 2018 AND LANGUAGE(english)
6. 3 and 4 and 5
7. TITLE-ABS-KEY(newborn* or neonat* or infant* or toddler* or child* or adolescent* or pediatric* or pediatric* or girl or girls or boy or boys or teen or teens or teenager* or preschooler* or “pre-schooler*” or preteen or preteens or “pre-teen” or “pre-teens” or youth or youths) AND NOT TITLE-ABS-KEY(adult or adults or “middle age” or “middle aged” OR elderly OR geriatric* OR “old people” OR “old person*” OR “older people” OR “older person*” OR “very old”)
8. 6 and not 7
9. DOCTYPE(ab) OR DOCTYPE(ed) OR DOCTYPE(bk) OR DOCTYPE(er) OR DOCTYPE(no) OR DOCTYPE(sh)
10. 8 and not 9
11. INDEX(embase) OR INDEX(medline) OR PMID(0* OR 1* OR 2* OR 3* OR 4* OR 5* OR 6* OR 7* OR 8* OR 9*)
12. 10 and not 11

 © 2025. The Author(s).

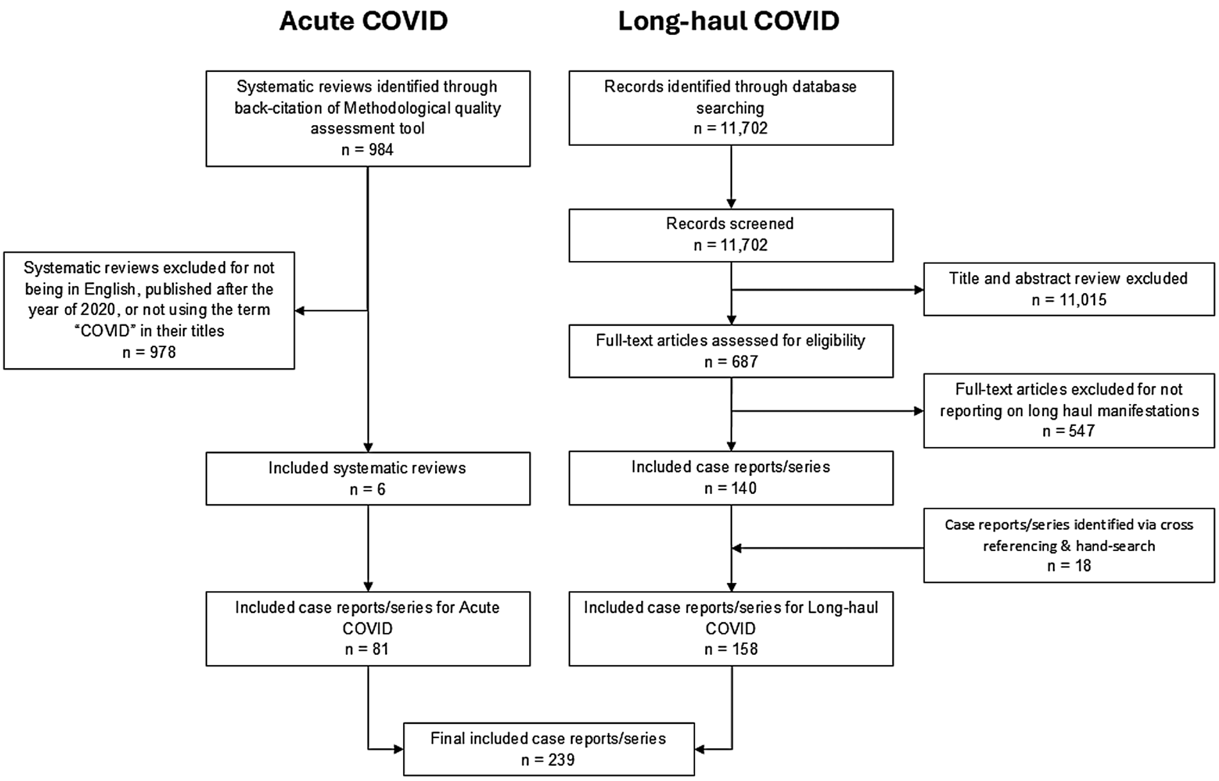

Supplementary Fig. S1 The process of study selection.

Supplementary Table S1 Bibliography of included case reports/series

1. Abdallah, H.; Porterfield, F.; Fajgenbaum, D., Symptomatic relapse and long-term sequelae of COVID-19 in a previously healthy 30-year-old man. *BMJ Case Rep* **2020,** *13* (12), e239825.
2. Buselli, R.; Corsi, M.; Necciari, G.; Pistolesi, P.; Baldanzi, S.; Chiumiento, M.; Del Lupo, E.; Guerra, P. D.; Cristaudo, A., Sudden and persistent dysphonia within the framework of COVID-19: The case report of a nurse. *Brain Behav Immun Health* **2020,** *9*, 100160.
3. Dani, M.; Dirksen, A.; Taraborrelli, P.; Torocastro, M.; Panagopoulos, D.; Sutton, R.; Lim, P. B., Autonomic dysfunction in 'long COVID': rationale, physiology and management strategies. *Clin Med (Lond)* **2021,** *21* (1), e63-e67.
4. Guedj, E.; Million, M.; Dudouet, P.; Tissot-Dupont, H.; Bregeon, F.; Cammilleri, S.; Raoult, D., (18)F-FDG brain PET hypometabolism in post-SARS-CoV-2 infection: substrate for persistent/delayed disorders? *Eur J Nucl Med Mol Imaging* **2021,** *48* (2), 592–595.
5. Hellmuth, J.; Barnett, T. A.; Asken, B. M.; Kelly, J. D.; Torres, L.; Stephens, M. L.; Greenhouse, B.; Martin, J. N.; Chow, F. C.; Deeks, S. G.; Greene, M.; Miller, B. L.; Annan, W.; Henrich, T. J.; Peluso, M. J., Persistent COVID-19-associated neurocognitive symptoms in non-hospitalized patients. *J Neurovirol* **2021,** *27* (1), 191–195.
6. Heiss, R.; Grodzki, D. M.; Horger, W.; Uder, M.; Nagel, A. M.; Bickelhaupt, S., High-performance low field MRI enables visualization of persistent pulmonary damage after COVID-19. *Magn Reson Imaging* **2021,** *76*, 49–51.
7. Hosseini, Z.; Ghodsi, S.; Hejazi, S. F., Persistent Complete Heart Block in a Patient with COVID-19 Infection: a Case Report. *SN Compr Clin Med* **2021,** *3* (1), 259–262.
8. Lim, S. T.; Janaway, B.; Costello, H.; Trip, A.; Price, G., Persistent psychotic symptoms following COVID-19 infection. *BJPsych Open* **2020,** *6* (5), e105.
9. Ludvigsson, J. F., Reporting suspicions of long COVID in children is justified during this global emergency. *Acta Paediatr* **2021,** *110* (4), 1373.
10. Manckoundia, P.; Franon, E., Is Persistent Thick Copious Mucus a Long-Term Symptom of COVID-19? *Eur J Case Rep Intern Med* **2020,** *7* (12), 002145.
11. Negrini, F.; Ferrario, I.; Mazziotti, D.; Berchicci, M.; Bonazzi, M.; de Sire, A.; Negrini, S.; Zapparoli, L., Neuropsychological Features of Severe Hospitalized Coronavirus Disease 2019 Patients at Clinical Stability and Clues for Postacute Rehabilitation. *Arch Phys Med Rehabil* **2021,** *102* (1), 155–158.
12. Novak, P., Post COVID-19 syndrome associated with orthostatic cerebral hypoperfusion syndrome, small fiber neuropathy and benefit of immunotherapy: a case report. *eNeurologicalSci* **2020,** *21*, 100276.
13. Raahimi, M. M.; Kane, A.; Moore, C. E.; Alareed, A. W., Late onset of Guillain-Barre syndrome following SARS-CoV-2 infection: part of 'long COVID-19 syndrome'? *BMJ Case Rep* **2021,** *14* (1), e240178.
14. Sampaio Rocha-Filho, P. A.; Voss, L., Persistent Headache and Persistent Anosmia Associated With COVID-19. *Headache* **2020,** *60* (8), 1797–1799.

 © 2025. The Author(s).

Supplementary Table S1 (*Continued*)

15. Medina, L. C. G., Telogen Effluvium and Alopecia Areata as Clinical Manifestations of Covid-19. *Research Review* **2022.**
16. Clouden, T. A., Persistent Hallucinations in a 46-Year-Old Woman After COVID-19 Infection: A Case Report. *Cureus* **2020,** *12* (12), e11993.
17. Yao, X. H.; He, Z. C.; Li, T. Y.; Zhang, H. R.; Wang, Y.; Mou, H.; Guo, Q.; Yu, S. C.; Ding, Y.; Liu, X.; Ping, Y. F.; Bian, X. W., Pathological evidence for residual SARS-CoV-2 in pulmonary tissues of a ready-for-discharge patient. *Cell Res* **2020,** *30* (6), 541–543.
18. Zhu, M.; Chen, D.; Zhu, Y.; Xiong, X.; Ding, Y.; Guo, F.; Zhu, M.; Zhou, J., Long-term sero-positivity for IgG, sequelae of respiratory symptoms, and abundance of malformed sperms in a patient recovered from severe COVID-19. *Eur J Clin Microbiol Infect Dis* **2021,** *40* (7), 1559–1567.
19. Bonomi, L.; Ghilardi, L.; Arnoldi, E.; Tondini, C. A.; Bettini, A. C., A Rapid Fatal Evolution of Coronavirus Disease-19 in a Patient With Advanced Lung Cancer With a Long-Time Response to Nivolumab. *J Thorac Oncol* **2020,** *15* (6), e83-e85.
20. Cai, Y.; Hao, Z.; Gao, Y.; Ping, W.; Wang, Q.; Peng, S.; Zhao, B.; Sun, W.; Zhu, M.; Li, K.; Han, Y.; Kuang, D.; Chu, Q.; Fu, X.; Zhang, N., Coronavirus Disease 2019 in the Perioperative Period of Lung Resection: A Brief Report From a Single Thoracic Surgery Department in Wuhan, People's Republic of China. *J Thorac Oncol* **2020,** *15* (6), 1065–1072.
21. Guan, W. J.; Liang, W. H.; Zhao, Y.; Liang, H. R.; Chen, Z. S.; Li, Y. M.; Liu, X. Q.; Chen, R. C.; Tang, C. L.; Wang, T.; Ou, C. Q.; Li, L.; Chen, P. Y.; Sang, L.; Wang, W.; Li, J. F.; Li, C. C.; Ou, L. M.; Cheng, B.; Xiong, S.; Ni, Z. Y.; Xiang, J.; Hu, Y.; Liu, L.; Shan, H.; Lei, C. L.; Peng, Y. X.; Wei, L.; Liu, Y.; Hu, Y. H.; Peng, P.; Wang, J. M.; Liu, J. Y.; Chen, Z.; Li, G.; Zheng, Z. J.; Qiu, S. Q.; Luo, J.; Ye, C. J.; Zhu, S. Y.; Cheng, L. L.; Ye, F.; Li, S. Y.; Zheng, J. P.; Zhang, N. F.; Zhong, N. S.; He, J. X.; China Medical Treatment Expert Group for, C., Comorbidity and its impact on 1590 patients with COVID-19 in China: a nationwide analysis. *Eur Respir J* **2020,** *55* (5), 2000547.
22. Huang, C.; Wang, Y.; Li, X.; Ren, L.; Zhao, J.; Hu, Y.; Zhang, L.; Fan, G.; Xu, J.; Gu, X.; Cheng, Z.; Yu, T.; Xia, J.; Wei, Y.; Wu, W.; Xie, X.; Yin, W.; Li, H.; Liu, M.; Xiao, Y.; Gao, H.; Guo, L.; Xie, J.; Wang, G.; Jiang, R.; Gao, Z.; Jin, Q.; Wang, J.; Cao, B., Clinical features of patients infected with 2019 novel coronavirus in Wuhan, China. *Lancet* **2020,** *395* (10223), 497–506.
23. Jazieh, A. R.; Alenazi, T. H.; Alhejazi, A.; Al Safi, F.; Al Olayan, A., Outcome of Oncology Patients Infected With Coronavirus. *JCO Glob Oncol* **2020,** *6*, 471–475.
24. Liang, W.; Guan, W.; Chen, R.; Wang, W.; Li, J.; Xu, K.; Li, C.; Ai, Q.; Lu, W.; Liang, H.; Li, S.; He, J., Cancer patients in SARS-CoV-2 infection: a nationwide analysis in China. *Lancet Oncol* **2020,** *21* (3), 335–337.
25. Qin, J.; Wang, H.; Qin, X.; Zhang, P.; Zhu, L.; Cai, J.; Yuan, Y.; Li, H., Perioperative Presentation of COVID-19 Disease in a Liver Transplant Recipient. *Hepatology* **2020,** *72* (4), 1491–1493.
26. Spezzani, V.; Piunno, A.; Iselin, H. U., Benign COVID-19 in an immunocompromised cancer patient - the case of a married couple. *Swiss Med Wkly* **2020,** *150*, w20246.
27. Tian, S.; Hu, W.; Niu, L.; Liu, H.; Xu, H.; Xiao, S. Y., Pulmonary Pathology of Early-Phase 2019 Novel Coronavirus (COVID-19) Pneumonia in Two Patients With Lung Cancer. *J Thorac Oncol* **2020,** *15* (5), 700–704.
28. Yu, J.; Ouyang, W.; Chua, M. L. K.; Xie, C., SARS-CoV-2 Transmission in Patients With Cancer at a Tertiary Care Hospital in Wuhan, China. *JAMA Oncol* **2020,** *6* (7), 1108–1110.
29. Zhang, H.; Xie, C.; Huang, Y., Treatment and Outcome of a Patient With Lung Cancer Infected With Severe Acute Respiratory Syndrome Coronavirus-2. *J Thorac Oncol* **2020,** *15* (5), e63-e64.
30. Zhang, L.; Zhu, F.; Xie, L.; Wang, C.; Wang, J.; Chen, R.; Jia, P.; Guan, H. Q.; Peng, L.; Chen, Y.; Peng, P.; Zhang, P.; Chu, Q.; Shen, Q.; Wang, Y.; Xu, S. Y.; Zhao, J. P.; Zhou, M., Clinical characteristics of COVID-19-infected cancer patients: a retrospective case study in three hospitals within Wuhan, China. *Ann Oncol* **2020,** *31* (7), 894–901.
31. Zhang, X.; Song, K.; Tong, F.; Fei, M.; Guo, H.; Lu, Z.; Wang, J.; Zheng, C., First case of COVID-19 in a patient with multiple myeloma successfully treated with tocilizumab. *Blood Adv* **2020,** *4* (7), 1307–1310.
32. Wei, J.; Xu, H.; Xiong, J.; Shen, Q.; Fan, B.; Ye, C.; Dong, W.; Hu, F., 2019 Novel Coronavirus (COVID-19) Pneumonia: Serial Computed Tomography Findings. *Korean J Radiol* **2020,** *21* (4), 501–504.
33. Shi, H.; Han, X.; Zheng, C., Evolution of CT Manifestations in a Patient Recovered from 2019 Novel Coronavirus (2019-nCoV) Pneumonia in Wuhan, China. *Radiology* **2020,** *295* (1), 20.
34. Holshue, M. L.; DeBolt, C.; Lindquist, S.; Lofy, K. H.; Wiesman, J.; Bruce, H.; Spitters, C.; Ericson, K.; Wilkerson, S.; Tural, A.; Diaz, G.; Cohn, A.; Fox, L.; Patel, A.; Gerber, S. I.; Kim, L.; Tong, S.; Lu, X.; Lindstrom, S.; Pallansch, M. A.; Weldon, W. C.; Biggs, H. M.; Uyeki, T. M.; Pillai, S. K.; Washington State -nCo, V. C. I. T., First Case of 2019 Novel Coronavirus in the United States. *N Engl J Med* **2020,** *382* (10), 929–936.
35. Chen, D.; Xu, W.; Lei, Z.; Huang, Z.; Liu, J.; Gao, Z.; Peng, L., Recurrence of positive SARS-CoV-2 RNA in COVID-19: A case report. *Int J Infect Dis* **2020,** *93*, 297–299.
36. Cheng, S. C.; Chang, Y. C.; Fan Chiang, Y. L.; Chien, Y. C.; Cheng, M.; Yang, C. H.; Huang, C. H.; Hsu, Y. N., First case of Coronavirus Disease 2019 (COVID-19) pneumonia in Taiwan. *J Formos Med Assoc* **2020,** *119* (3), 747–751.
37. Huang, W. H.; Teng, L. C.; Yeh, T. K.; Chen, Y. J.; Lo, W. J.; Wu, M. J.; Chin, C. S.; Tsan, Y. T.; Lin, T. C.; Chai, J. W.; Lin, C. F.; Tseng, C. H.; Liu, C. W.; Wu, C. M.; Chen, P. Y.; Shi, Z. Y.; Liu, P. Y., 2019 novel coronavirus disease (COVID-19) in Taiwan: Reports of two cases from Wuhan, China. *J Microbiol Immunol Infect* **2020,** *53* (3), 481–484.
38. Lescure, F. X.; Bouadma, L.; Nguyen, D.; Parisey, M.; Wicky, P. H.; Behillil, S.; Gaymard, A.; Bouscambert-Duchamp, M.; Donati, F.; Le Hingrat, Q.; Enouf, V.; Houhou-Fidouh, N.; Valette, M.; Mailles, A.; Lucet, J. C.; Mentre, F.; Duval, X.; Descamps, D.; Malvy, D.; Timsit, J. F.; Lina, B.; van-der-Werf, S.; Yazdanpanah, Y., Clinical and virological data of the first cases of COVID-19 in Europe: a case series. *Lancet Infect Dis* **2020,** *20* (6), 697–706.
39. Han, W.; Quan, B.; Guo, Y.; Zhang, J.; Lu, Y.; Feng, G.; Wu, Q.; Fang, F.; Cheng, L.; Jiao, N.; Li, X.; Chen, Q., The course of clinical diagnosis and treatment of a case infected with coronavirus disease 2019. *J Med Virol* **2020,** *92* (5), 461–463.
40. Kim, J. Y.; Choe, P. G.; Oh, Y.; Oh, K. J.; Kim, J.; Park, S. J.; Park, J. H.; Na, H. K.; Oh, M. D., The First Case of 2019 Novel Coronavirus Pneumonia Imported into Korea from Wuhan, China: Implication for Infection Prevention and Control Measures. *J Korean Med Sci* **2020,** *35* (5), e61.

(*Continued*)

 © 2025. The Author(s).

Supplementary Table S1 (*Continued*)

41. Xu, Z.; Shi, L.; Wang, Y.; Zhang, J.; Huang, L.; Zhang, C.; Liu, S.; Zhao, P.; Liu, H.; Zhu, L.; Tai, Y.; Bai, C.; Gao, T.; Song, J.; Xia, P.; Dong, J.; Zhao, J.; Wang, F. S., Pathological findings of COVID-19 associated with acute respiratory distress syndrome. *Lancet Respir Med* **2020,** *8* (4), 420–422.
42. Lan, L.; Xu, D.; Ye, G.; Xia, C.; Wang, S.; Li, Y.; Xu, H., Positive RT-PCR Test Results in Patients Recovered From COVID-19. *JAMA* **2020,** *323* (15), 1502–1503.
43. Shen, C.; Wang, Z.; Zhao, F.; Yang, Y.; Li, J.; Yuan, J.; Wang, F.; Li, D.; Yang, M.; Xing, L.; Wei, J.; Xiao, H.; Yang, Y.; Qu, J.; Qing, L.; Chen, L.; Xu, Z.; Peng, L.; Li, Y.; Zheng, H.; Chen, F.; Huang, K.; Jiang, Y.; Liu, D.; Zhang, Z.; Liu, Y.; Liu, L., Treatment of 5 Critically Ill Patients With COVID-19 With Convalescent Plasma. *JAMA* **2020,** *323* (16), 1582–1589.
44. Inciardi, R. M.; Lupi, L.; Zaccone, G.; Italia, L.; Raffo, M.; Tomasoni, D.; Cani, D. S.; Cerini, M.; Farina, D.; Gavazzi, E.; Maroldi, R.; Adamo, M.; Ammirati, E.; Sinagra, G.; Lombardi, C. M.; Metra, M., Cardiac Involvement in a Patient With Coronavirus Disease 2019 (COVID-19). *JAMA Cardiol* **2020,** *5* (7), 819–824.
45. Ferrey, A. J.; Choi, G.; Hanna, R. M.; Chang, Y.; Tantisattamo, E.; Ivaturi, K.; Park, E.; Nguyen, L.; Wang, B.; Tonthat, S.; Rhee, C. M.; Reddy, U.; Lau, W. L.; Huang, S. S.; Gohil, S.; Amin, A. N.; Hsieh, L.; Cheng, T. T.; Lee, R. A.; Kalantar-Zadeh, K., A Case of Novel Coronavirus Disease 19 in a Chronic Hemodialysis Patient Presenting with Gastroenteritis and Developing Severe Pulmonary Disease. *Am J Nephrol* **2020,** *51* (5), 337–342.
46. Michot, J. M.; Albiges, L.; Chaput, N.; Saada, V.; Pommeret, F.; Griscelli, F.; Balleyguier, C.; Besse, B.; Marabelle, A.; Netzer, F.; Merad, M.; Robert, C.; Barlesi, F.; Gachot, B.; Stoclin, A., Tocilizumab, an anti-IL-6 receptor antibody, to treat COVID-19-related respiratory failure: a case report. *Ann Oncol* **2020,** *31* (7), 961–964.
47. Mihai, C.; Dobrota, R.; Schroder, M.; Garaiman, A.; Jordan, S.; Becker, M. O.; Maurer, B.; Distler, O., COVID-19 in a patient with systemic sclerosis treated with tocilizumab for SSc-ILD. *Ann Rheum Dis* **2020,** *79* (5), 668–669.
48. Durante-Mangoni, E.; Andini, R.; Bertolino, L.; Mele, F.; Florio, L. L.; Murino, P.; Corcione, A.; Zampino, R., Early experience with remdesivir in SARS-CoV-2 pneumonia. *Infection* **2020,** *48* (5), 779–782.
49. Lim, J.; Jeon, S.; Shin, H. Y.; Kim, M. J.; Seong, Y. M.; Lee, W. J.; Choe, K. W.; Kang, Y. M.; Lee, B.; Park, S. J., Case of the Index Patient Who Caused Tertiary Transmission of COVID-19 Infection in Korea: the Application of Lopinavir/Ritonavir for the Treatment of COVID-19 Infected Pneumonia Monitored by Quantitative RT-PCR. *J Korean Med Sci* **2020,** *35* (6), e79.
50. Chen, R.; Zhang, Y.; Huang, L.; Cheng, B. H.; Xia, Z. Y.; Meng, Q. T., Safety and efficacy of different anesthetic regimens for parturients with COVID-19 undergoing Cesarean delivery: a case series of 17 patients. *Can J Anaesth* **2020,** *67* (6), 655–663.
51. Liu, D.; Li, L.; Wu, X.; Zheng, D.; Wang, J.; Yang, L.; Zheng, C., Pregnancy and Perinatal Outcomes of Women With Coronavirus Disease (COVID-19) Pneumonia: A Preliminary Analysis. *AJR Am J Roentgenol* **2020,** *215* (1), 127–132.
52. Zeng, L.; Xia, S.; Yuan, W.; Yan, K.; Xiao, F.; Shao, J.; Zhou, W., Neonatal Early-Onset Infection With SARS-CoV-2 in 33 Neonates Born to Mothers With COVID-19 in Wuhan, China. *JAMA Pediatr* **2020,** *174* (7), 722–725.
53. Nie, R.; Wang, S.-s.; Yang, Q.; Fan, C.-f.; Liu, Y.-l.; He, W.-c.; Jiang, M.; Liu, C.-c.; Zeng, W.-j.; Wu, J.-l.; Oktay, K.; Feng, L.; Jin, L., Clinical features and the maternal and neonatal outcomes of pregnant women with coronavirus disease 2019. Cold Spring Harbor Laboratory: 2020.
54. Zhang, Y.; Chen, R.; Wang, J.; Gong, Y.; Zhou, Q.; Cheng, H.-h.; Xia, Z.-y.; Chen, X.; Meng, Q.-t.; Ma, D., Anesthetic managment and clinical outcomes of parturients with COVID-19: a multicentre, retrospective, propensity score matched cohort study. Cold Spring Harbor Laboratory: 2020.
55. Ferrazzi, E. M.; Frigerio, L.; Cetin, I.; Vergani, P.; Spinillo, A.; Prefumo, F.; Pellegrini, E.; Gargantini, G., COVID-19 Obstetrics Task Force, Lombardy, Italy: Executive management summary and short report of outcome. *Int J Gynaecol Obstet* **2020,** *149* (3), 377–378.
56. Khan, S.; Jun, L.; Nawsherwan; Siddique, R.; Li, Y.; Han, G.; Xue, M.; Nabi, G.; Liu, J., Association of COVID-19 with pregnancy outcomes in health-care workers and general women. *Clin Microbiol Infect* **2020,** *26* (6), 788–790.
57. Wu, X.; Sun, R.; Chen, J.; Xie, Y.; Zhang, S.; Wang, X., Radiological findings and clinical characteristics of pregnant women with COVID-19 pneumonia. *Int J Gynaecol Obstet* **2020,** *150* (1), 58–63.
58. Breslin, N.; Baptiste, C.; Gyamfi-Bannerman, C.; Miller, R.; Martinez, R.; Bernstein, K.; Ring, L.; Landau, R.; Purisch, S.; Friedman, A. M.; Fuchs, K.; Sutton, D.; Andrikopoulou, M.; Rupley, D.; Sheen, J. J.; Aubey, J.; Zork, N.; Moroz, L.; Mourad, M.; Wapner, R.; Simpson, L. L.; D'Alton, M. E.; Goffman, D., Coronavirus disease 2019 infection among asymptomatic and symptomatic pregnant women: two weeks of confirmed presentations to an affiliated pair of New York City hospitals. *Am J Obstet Gynecol MFM* **2020,** *2* (2), 100118.
59. Sutton, D.; Fuchs, K.; D'Alton, M.; Goffman, D., Universal Screening for SARS-CoV-2 in Women Admitted for Delivery. *N Engl J Med* **2020,** *382* (22), 2163–2164.
60. Liu, W.; Wang, J.; Li, W.; Zhou, Z.; Liu, S.; Rong, Z., Clinical characteristics of 19 neonates born to mothers with COVID-19. *Front Med* **2020,** *14* (2), 193–198.
61. Chen, L.; Li, Q.; Zheng, D.; Jiang, H.; Wei, Y.; Zou, L.; Feng, L.; Xiong, G.; Sun, G.; Wang, H.; Zhao, Y.; Qiao, J., Clinical Characteristics of Pregnant Women with Covid-19 in Wuhan, China. *N Engl J Med* **2020,** *382* (25), e100.
62. Yan, J.; Guo, J.; Fan, C.; Juan, J.; Yu, X.; Li, J.; Feng, L.; Li, C.; Chen, H.; Qiao, Y.; Lei, D.; Wang, C.; Xiong, G.; Xiao, F.; He, W.; Pang, Q.; Hu, X.; Wang, S.; Chen, D.; Zhang, Y.; Poon, L. C.; Yang, H., Coronavirus disease 2019 in pregnant women: a report based on 116 cases. *Am J Obstet Gynecol* **2020,** *223* (1), 111 e1–111 e14.
63. Chen, H.; Guo, J.; Wang, C.; Luo, F.; Yu, X.; Zhang, W.; Li, J.; Zhao, D.; Xu, D.; Gong, Q.; Liao, J.; Yang, H.; Hou, W.; Zhang, Y., Clinical characteristics and intrauterine vertical transmission potential of COVID-19 infection in nine pregnant women: a retrospective review of medical records. *Lancet* **2020,** *395* (10226), 809–815.
64. Chen, Y.; Peng, H.; Wang, L.; Zhao, Y.; Zeng, L.; Gao, H.; Liu, Y., Infants Born to Mothers With a New Coronavirus (COVID-19). *Front Pediatr* **2020,** *8*, 104.
65. Dong, L.; Tian, J.; He, S.; Zhu, C.; Wang, J.; Liu, C.; Yang, J., Possible Vertical Transmission of SARS-CoV-2 From an Infected Mother to Her Newborn. *JAMA* **2020,** *323* (18), 1846–1848.
66. Gidlof, S.; Savchenko, J.; Brune, T.; Josefsson, H., COVID-19 in pregnancy with comorbidities: More liberal testing strategy is needed. *Acta Obstet Gynecol Scand* **2020,** *99* (7), 948–949.

 © 2025. The Author(s).

Supplementary Table S1 (*Continued*)

67. Huang, J. W.; Zhou, X. Y.; Lu, S. J.; Xu, Y.; Hu, J. B.; Huang, M. L.; Wang, H. F.; Hu, C. C.; Li, S. G.; Chen, J. K.; Wang, Z.; Hu, S. H.; Wei, N., Dialectical behavior therapy-based psychological intervention for woman in late pregnancy and early postpartum suffering from COVID-19: a case report. *J Zhejiang Univ Sci B* **2020,** *21* (5), 394–399.
68. Iqbal, S. N.; Overcash, R.; Mokhtari, N.; Saeed, H.; Gold, S.; Auguste, T.; Mirza, M. U.; Ruiz, M. E.; Chahine, J. J.; Waga, M.; Wortmann, G., An Uncomplicated Delivery in a Patient with Covid-19 in the United States. *N Engl J Med* **2020,** *382* (16), e34.
69. Kalafat, E.; Yaprak, E.; Cinar, G.; Varli, B.; Ozisik, S.; Uzun, C.; Azap, A.; Koc, A., Lung ultrasound and computed tomographic findings in pregnant woman with COVID-19. *Ultrasound Obstet Gynecol* **2020,** *55* (6), 835–837.
70. Khan, S.; Peng, L.; Siddique, R.; Nabi, G.; Nawsherwan; Xue, M.; Liu, J.; Han, G., Impact of COVID-19 infection on pregnancy outcomes and the risk of maternal-to-neonatal intrapartum transmission of COVID-19 during natural birth. *Infect Control Hosp Epidemiol* **2020,** *41* (6), 748–750.
71. Lee, D. H.; Lee, J.; Kim, E.; Woo, K.; Park, H. Y.; An, J., Emergency cesarean section performed in a patient with confirmed severe acute respiratory syndrome Coronavirus-2 -a case report. *Korean J Anesthesiol* **2020,** *73* (4), 347–351.
72. Li, Y.; Zhao, R.; Zheng, S.; Chen, X.; Wang, J.; Sheng, X.; Zhou, J.; Cai, H.; Fang, Q.; Yu, F.; Fan, J.; Xu, K.; Chen, Y.; Sheng, J., Lack of Vertical Transmission of Severe Acute Respiratory Syndrome Coronavirus 2, China. *Emerg Infect Dis* **2020,** *26* (6), 1335–1336.
73. Liao, X.; Yang, H.; Kong, J.; Yang, H., Chest CT Findings in a Pregnant Patient with 2019 Novel Coronavirus Disease. *Balkan Med J* **2020,** *37* (4), 226–228.
74. Liu, J.; Lian, R.; Zhang, G.; Hou, B.; Wang, C.; Dong, J.; Yang, L.; Wang, J.; Dai, S.; Chen, L.; Zhang, G.; Lu, X., IgM/IgG antibody changes in asymptomatic and discharged patients with reoccurring positive nucleic acid test (RP) of Novel Coronavirus Disease 2019 (COVID-19). Authorea, Inc.: 2020.
75. Liu, Y.; Chen, H.; Tang, K.; Guo, Y., Withdrawn: Clinical manifestations and outcome of SARS-CoV-2 infection during pregnancy. *J Infect* **2020**, S0163–4453(20)30109–2.
76. Wang, S.; Guo, L.; Chen, L.; Liu, W.; Cao, Y.; Zhang, J.; Feng, L., A Case Report of Neonatal 2019 Coronavirus Disease in China. *Clin Infect Dis* **2020,** *71* (15), 853–857.
77. Wang, X.; Zhou, Z.; Zhang, J.; Zhu, F.; Tang, Y.; Shen, X., A Case of 2019 Novel Coronavirus in a Pregnant Woman With Preterm Delivery. *Clin Infect Dis* **2020,** *71* (15), 844–846.
78. Xiong, X.; Wei, H.; Zhang, Z.; Chang, J.; Ma, X.; Gao, X.; Chen, Q.; Pang, Q., Vaginal delivery report of a healthy neonate born to a convalescent mother with COVID–19. *J Med Virol* **2020,** *92* (9), 1657–1659.
79. Zamaniyan, M.; Ebadi, A.; Mir, S. A.; Rahmani, Z.; Haghshenas, M.; Azizi, S., Iatrogenic Preterm delivery in pregnant woman with critical COVID-19 pneumonia and vertical transmission: A case report. In *Authorea*, Authorea, Inc.
80. Zeng, H.; Xu, C.; Fan, J.; Tang, Y.; Deng, Q.; Zhang, W.; Long, X., Antibodies in Infants Born to Mothers With COVID-19 Pneumonia. *JAMA* **2020,** *323* (18), 1848–1849.
81. Zhu, H.; Wang, L.; Fang, C.; Peng, S.; Zhang, L.; Chang, G.; Xia, S.; Zhou, W., Clinical analysis of 10 neonates born to mothers with 2019-nCoV pneumonia. *Transl Pediatr* **2020,** *9* (1), 51–60.
82. Wang, D.; Hu, B.; Hu, C.; Zhu, F.; Liu, X.; Zhang, J.; Wang, B.; Xiang, H.; Cheng, Z.; Xiong, Y.; Zhao, Y.; Li, Y.; Wang, X.; Peng, Z., Clinical Characteristics of 138 Hospitalized Patients With 2019 Novel Coronavirus-Infected Pneumonia in Wuhan, China. *JAMA* **2020,** *323* (11), 1061–1069.
83. Kaushik, S.; Aydin, S. I.; Derespina, K. R.; Bansal, P. B.; Kowalsky, S.; Trachtman, R.; Gillen, J. K.; Perez, M. M.; Soshnick, S. H.; Conway, E. E., Jr.; Bercow, A.; Seiden, H. S.; Pass, R. H.; Ushay, H. M.; Ofori-Amanfo, G.; Medar, S. S., Multisystem Inflammatory Syndrome in Children Associated with Severe Acute Respiratory Syndrome Coronavirus 2 Infection (MIS-C): A Multi-institutional Study from New York City. *J Pediatr* **2020,** *224*, 24–29.
84. Riollano-Cruz, M.; Akkoyun, E.; Briceno-Brito, E.; Kowalsky, S.; Reed, J.; Posada, R.; Sordillo, E. M.; Tosi, M.; Trachtman, R.; Paniz-Mondolfi, A., Multisystem inflammatory syndrome in children related to COVID-19: A New York City experience. *J Med Virol* **2021,** *93* (1), 424–433.
85. Chiotos, K.; Bassiri, H.; Behrens, E. M.; Blatz, A. M.; Chang, J.; Diorio, C.; Fitzgerald, J. C.; Topjian, A.; John, A. R. O., Multisystem Inflammatory Syndrome in Children During the Coronavirus 2019 Pandemic: A Case Series. *J Pediatric Infect Dis Soc* **2020,** *9* (3), 393–398.
86. Capone, C. A.; Subramony, A.; Sweberg, T.; Schneider, J.; Shah, S.; Rubin, L.; Schleien, C.; Northwell Health, C.-R. C.; Epstein, S.; Johnson, J. C.; Kessel, A.; Misra, N.; Mitchell, E.; Palumbo, N.; Rajan, S.; Rocker, J.; Williamson, K.; Davidson, K. W., Characteristics, Cardiac Involvement, and Outcomes of Multisystem Inflammatory Syndrome of Childhood Associated with severe acute respiratory syndrome coronavirus 2 Infection. *J Pediatr* **2020,** *224*, 141–145.
87. Greene, A. G.; Saleh, M.; Roseman, E.; Sinert, R., Toxic shock-like syndrome and COVID-19: Multisystem inflammatory syndrome in children (MIS-C). *Am J Emerg Med* **2020,** *38* (11), 2492 e5–2492 e6.
88. Dufort, E. M.; Koumans, E. H.; Chow, E. J.; Rosenthal, E. M.; Muse, A.; Rowlands, J.; Barranco, M. A.; Maxted, A. M.; Rosenberg, E. S.; Easton, D.; Udo, T.; Kumar, J.; Pulver, W.; Smith, L.; Hutton, B.; Blog, D.; Zucker, H.; New York, S.; Centers for Disease, C.; Prevention Multisystem Inflammatory Syndrome in Children Investigation, T., Multisystem Inflammatory Syndrome in Children in New York State. *N Engl J Med* **2020,** *383* (4), 347–358.
89. Miller, J.; Cantor, A.; Zachariah, P.; Ahn, D.; Martinez, M.; Margolis, K. G., Gastrointestinal Symptoms as a Major Presentation Component of a Novel Multisystem Inflammatory Syndrome in Children That Is Related to Coronavirus Disease 2019: A Single Center Experience of 44 Cases. *Gastroenterology* **2020,** *159* (4), 1571–1574 e2.
90. Lee, P. Y.; Day-Lewis, M.; Henderson, L. A.; Friedman, K. G.; Lo, J.; Roberts, J. E.; Lo, M. S.; Platt, C. D.; Chou, J.; Hoyt, K. J.; Baker, A. L.; Banzon, T. M.; Chang, M. H.; Cohen, E.; de Ferranti, S. D.; Dionne, A.; Habiballah, S.; Halyabar, O.; Hausmann, J. S.; Hazen, M. M.; Janssen, E.; Meidan, E.; Nelson, R. W.; Nguyen, A. A.; Sundel, R. P.; Dedeoglu, F.; Nigrovic, P. A.; Newburger, J. W.; Son, M. B. F., Distinct clinical and immunological features of SARS-CoV-2-induced multisystem inflammatory syndrome in children. *J Clin Invest* **2020,** *130* (11), 5942–5950.
91. Belot, A.; Antona, D.; Renolleau, S.; Javouhey, E.; Hentgen, V.; Angoulvant, F.; Delacourt, C.; Iriart, X.; Ovaert, C.; Bader-Meunier, B.; Kone-Paut, I.; Levy-Bruhl, D., SARS-CoV-2-related pediatric inflammatory multisystem syndrome, an epidemiological study, France, 1 March to 17 May 2020. *Euro Surveill* **2020,** *25* (22), 2001010.

(*Continued*)

 © 2025. The Author(s).

Supplementary Table S1 (*Continued*)

92. Verdoni, L.; Mazza, A.; Gervasoni, A.; Martelli, L.; Ruggeri, M.; Ciuffreda, M.; Bonanomi, E.; D'Antiga, L., An outbreak of severe Kawasaki-like disease at the Italian epicentre of the SARS-CoV-2 epidemic: an observational cohort study. *Lancet* **2020**, *395* (10239), 1771–1778.
93. Balasubramanian, S.; Nagendran, T. M.; Ramachandran, B.; Ramanan, A. V., Hyper-inflammatory Syndrome in a Child With COVID-19 Treated Successfully With Intravenous Immunoglobulin and Tocilizumab. *Indian Pediatr* **2020,** *57* (7), 681–683.
94. Rauf, A.; Vijayan, A.; John, S. T.; Krishnan, R.; Latheef, A., Multisystem Inflammatory Syndrome with Features of Atypical Kawasaki Disease during COVID-19 Pandemic. *Indian J Pediatr* **2020,** *87* (9), 745–747.
95. Acharyya, B. C.; Acharyya, S.; Das, D., Novel Coronavirus Mimicking Kawasaki Disease in an Infant. *Indian Pediatr* **2020,** *57* (8), 753–754.
96. Whittaker, E.; Bamford, A.; Kenny, J.; Kaforou, M.; Jones, C. E.; Shah, P.; Ramnarayan, P.; Fraisse, A.; Miller, O.; Davies, P.; Kucera, F.; Brierley, J.; McDougall, M.; Carter, M.; Tremoulet, A.; Shimizu, C.; Herberg, J.; Burns, J. C.; Lyall, H.; Levin, M.; Group, P.-T. S.; Euclids; Consortia, P., Clinical Characteristics of 58 Children With a Pediatric Inflammatory Multisystem Syndrome Temporally Associated With SARS-CoV-2. *JAMA* **2020,** *324* (3), 259–269.
97. Moraleda, C.; Serna-Pascual, M.; Soriano-Arandes, A.; Simo, S.; Epalza, C.; Santos, M.; Grasa, C.; Rodriguez, M.; Soto, B.; Gallego, N.; Ruiz, Y.; Urretavizcaya-Martinez, M.; Pareja, M.; Sanz-Santaeufemia, F. J.; Fumado, V.; Lanaspa, M.; Jordan, I.; Prieto, L.; Belda, S.; Toral-Vazquez, B.; Rincon, E.; Gil-Villanueva, N.; Mendez-Echevarria, A.; Castillo-Serrano, A.; Riviere, J. G.; Soler-Palacin, P.; Rojo, P.; Tagarro, A.; Group, E.-A. W., Multi-inflammatory Syndrome in Children Related to Severe Acute Respiratory Syndrome Coronavirus 2 (SARS-CoV-2) in Spain. *Clin Infect Dis* **2021,** *72* (9), e397-e401.
98. Feldstein, L. R.; Rose, E. B.; Horwitz, S. M.; Collins, J. P.; Newhams, M. M.; Son, M. B. F.; Newburger, J. W.; Kleinman, L. C.; Heidemann, S. M.; Martin, A. A.; Singh, A. R.; Li, S.; Tarquinio, K. M.; Jaggi, P.; Oster, M. E.; Zackai, S. P.; Gillen, J.; Ratner, A. J.; Walsh, R. F.; Fitzgerald, J. C.; Keenaghan, M. A.; Alharash, H.; Doymaz, S.; Clouser, K. N.; Giuliano, J. S., Jr.; Gupta, A.; Parker, R. M.; Maddux, A. B.; Havalad, V.; Ramsingh, S.; Bukulmez, H.; Bradford, T. T.; Smith, L. S.; Tenforde, M. W.; Carroll, C. L.; Riggs, B. J.; Gertz, S. J.; Daube, A.; Lansell, A.; Coronado Munoz, A.; Hobbs, C. V.; Marohn, K. L.; Halasa, N. B.; Patel, M. M.; Randolph, A. G.; Overcoming, C.-I.; Team, C. C.-R., Multisystem Inflammatory Syndrome in U.S. Children and Adolescents. *N Engl J Med* **2020,** *383* (4), 334–346.
99. Kujawski, S. A.; Wong, K. K.; Collins, J. P.; Epstein, L.; Killerby, M. E.; Midgley, C. M.; Abedi, G. R.; Ahmed, N. S.; Almendares, O.; Alvarez, F. N.; Anderson, K. N.; Balter, S.; Barry, V.; Bartlett, K.; Beer, K.; Ben-Aderet, M. A.; Benowitz, I.; Biggs, H.; Binder, A. M.; Black, S. R.; Bonin, B.; Brown, C. M.; Bruce, H.; Bryant-Genevier, J.; Budd, A.; Buell, D.; Bystritsky, R.; Cates, J.; Charles, E. M.; Chatham-Stephens, K.; Chea, N.; Chiou, H.; Christiansen, D.; Chu, V.; Cody, S.; Cohen, M.; Conners, E.; Curns, A.; Dasari, V.; Dawson, P.; DeSalvo, T.; Diaz, G.; Donahue, M.; Donovan, S.; Duca, L. M.; Erickson, K.; Esona, M. D.; Evans, S.; Falk, J.; Feldstein, L. R.; Fenstersheib, M.; Fischer, M.; Fisher, R.; Foo, C.; Fricchione, M. J.; Friedman, O.; Fry, A. M.; Galang, R. R.; Garcia, M. M.; Gerber, S. I.; Gerrard, G.; Ghinai, I.; Gounder, P.; Grein, J.; Grigg, C.; Gunzenhauser, J. D.; Gutkin, G. I.; Haddix, M.; Hall, A. J.; Han, G.; Harcourt, J.; Harriman, K.; Haupt, T.; Haynes, A.; Holshue, M.; Hoover, C.; Hunter, J. C.; Jacobs, M. W.; Jarashow, C.; Jhung, M. A.; Joshi, K.; Kamali, T.; Kamili, S.; Kim, L.; Kim, M.; King, J.; Kirking, H. L.; Kita-Yarbro, A.; Klos, R.; Kobayashi, M.; Kocharian, A.; Komatsu, K. K.; Koppaka, R.; Layden, J. E.; Li, Y.; Lindquist, S.; Lindstrom, S.; Link-Gelles, R.; Lively, J.; Livingston, M.; Lo, K.; Lo, J.; Lu, X.; Lynch, B.; Madoff, L.; Malapati, L.; Marks, G.; Marlow, M.; Mathisen, G. E.; McClung, N.; McGovern, O.; McPherson, T. D.; Mehta, M.; Meier, A.; Mello, L.; Moon, S.-s.; Morgan, M.; Moro, R. N.; Murray, J.; Murthy, R.; Novosad, S.; Oliver, S. E.; O'Shea, J.; Pacilli, M.; Paden, C. R.; Pallansch, M. A.; Patel, M.; Patel, S.; Pedraza, I.; Pillai, S. K.; Pindyck, T.; Pray, I.; Queen, K.; Quick, N.; Reese, H.; Rha, B.; Rhodes, H.; Robinson, S.; Robinson, P.; Rolfes, M.; Routh, J.; Rubin, R.; Rudman, S. L.; Sakthivel, S. K.; Scott, S.; Shepherd, C.; Shetty, V.; Smith, E. A.; Smith, S.; Stierman, B.; Stoecker, W.; Sunenshine, R.; Sy-Santos, R.; Tamin, A.; Tao, Y.; Terashita, D.; Thornburg, N. J.; Tong, S.; Traub, E.; Tural, A.; Uehara, A.; Uyeki, T. M.; Vahey, G.; Verani, J. R.; Villarino, E.; Wallace, M.; Wang, L.; Watson, J. T.; Westercamp, M.; Whitaker, B.; Wilkerson, S.; Woodruff, R. C.; Wortham, J. M.; Wu, T.; Xie, A.; Yousaf, A.; Zahn, M.; Zhang, J., First 12 patients with coronavirus disease 2019 (COVID-19) in the United States. *medRxiv* **2020**, 2020.03.09.20032896.
100. Khatib, S.; Sabobeh, T.; Habib, A.; John, S.; Gomez, R.; Sivasankar, S.; Masoud, A., Post-COVID-19 fatigue as a major health problem: a cross-sectional study from Missouri, USA. *Ir J Med Sci* **2023,** *192* (2), 699–705.
101. AlRadini, F. A.; Alamri, F.; Aljahany, M. S.; Almuzaini, Y.; Alsofayan, Y.; Khan, A.; Albogami, N.; Abdulrahim, M.; Almogbil, A.; Alahmari, A., Post-acute COVID-19 condition in Saudi Arabia: A national representative study. *J Infect Public Health* **2022,** *15* (5), 526–532.
102. Yoo, S. M.; Liu, T. C.; Motwani, Y.; Sim, M. S.; Viswanathan, N.; Samras, N.; Hsu, F.; Wenger, N. S., Factors Associated with Post-Acute Sequelae of SARS-CoV-2 (PASC) After Diagnosis of Symptomatic COVID-19 in the Inpatient and Outpatient Setting in a Diverse Cohort. *J Gen Intern Med* **2022,** *37* (8), 1988–1995.
103. Iwashyna, T. J.; Kamphuis, L. A.; Gundel, S. J.; Hope, A. A.; Jolley, S.; Admon, A. J.; Caldwell, E.; Monahan, M. L.; Hauschildt, K.; Thompson, B. T.; Hough, C. L.; Prevention, N.; Early Treatment of Acute Lung Injury, N., Continuing Cardiopulmonary Symptoms, Disability, and Financial Toxicity 1 Month After Hospitalization for Third-Wave COVID-19: Early Results From a US Nationwide Cohort. *J Hosp Med* **2021,** *18*, 18.
104. Fernandez-de-Las-Penas, C.; Ryan-Murua, P.; Rodriguez-Jimenez, J.; Palacios-Cena, M.; Arendt-Nielsen, L.; Torres-Macho, J., Serological Biomarkers at Hospital Admission Are Not Related to Long-Term Post-COVID Fatigue and Dyspnea in COVID-19 Survivors. *Respiration* **2022,** *101* (7), 658–665.
105. Desgranges, F.; Tadini, E.; Munting, A.; Regina, J.; Filippidis, P.; Viala, B.; Karachalias, E.; Suttels, V.; Haefliger, D.; Kampouri, E.; Van Singer, M.; Tschopp, J.; Rochat Stettler, L.; Schaad, S.; Brahier, T.; Hugli, O.; Mueller, Y.; Gouveia, A.; Opota, O.; Carron, P. N.; Guery, B.; Papadimitriou-Olivgeris, M.; Boillat-Blanco, N.; Group, t. R. R., Post-COVID-19 Syndrome in Outpatients: a Cohort Study. *J Gen Intern Med* **2022,** *37* (8), 1943–1952.
106. Ferrucci, R.; Dini, M.; Rosci, C.; Capozza, A.; Groppo, E.; Reitano, M. R.; Allocco, E.; Poletti, B.; Brugnera, A.; Bai, F.; Monti, A.; Ticozzi, N.; Silani, V.; Centanni, S.; D'Arminio Monforte, A.; Tagliabue, L.; Priori, A., One-year cognitive follow-up of COVID-19 hospitalized patients. *Eur J Neurol* **2022,** *29* (7), 2006–2014.

 © 2025. The Author(s).

Supplementary Table S1 (*Continued*)

107. Liu, Y. H.; Chen, Y.; Wang, Q. H.; Wang, L. R.; Jiang, L.; Yang, Y.; Chen, X.; Li, Y.; Cen, Y.; Xu, C.; Zhu, J.; Li, W.; Wang, Y. R.; Zhang, L. L.; Liu, J.; Xu, Z. Q.; Wang, Y. J., One-Year Trajectory of Cognitive Changes in Older Survivors of COVID-19 in Wuhan, China: A Longitudinal Cohort Study. *JAMA Neurol* **2022,** *79* (5), 509–517.
108. Garout, M. A.; Saleh, S. A. K.; Adly, H. M.; Abdulkhaliq, A. A.; Khafagy, A. A.; Abdeltawab, M. R.; Rabaan, A. A.; Rodriguez-Morales, A. J.; Al-Tawfiq, J. A.; Alandiyjany, M. N., Post-COVID-19 syndrome: assessment of short- and long-term post-recovery symptoms in recovered cases in Saudi Arabia. *Infection* **2022,** *50* (6), 1431–1439.
109. Ferrando, S. J.; Dornbush, R.; Lynch, S.; Shahar, S.; Klepacz, L.; Karmen, C. L.; Chen, D.; Lobo, S. A.; Lerman, D., Neuropsychological, Medical, and Psychiatric Findings After Recovery From Acute COVID-19: A Cross-sectional Study. *J Acad Consult Liaison Psychiatry* **2022,** *63* (5), 474–484.
110. Forster, C.; Colombo, M. G.; Wetzel, A. J.; Martus, P.; Joos, S., Persisting Symptoms After COVID-19. *Dtsch Arztebl Int* **2022,** *119* (10), 167–174.
111. Zuschlag, D.; Grandt, D.; Custodis, F.; Braun, C.; Hauser, W., Spontaneously reported persistent symptoms related to coronavirus disease 2019 one year after hospital discharge: A retrospective cohort single-center study. *Schmerz* **2022,** *36* (5), 315–325.
112. Caspersen, I. H.; Magnus, P.; Trogstad, L., Excess risk and clusters of symptoms after COVID-19 in a large Norwegian cohort. *Eur J Epidemiol* **2022,** *37* (5), 539–548.
113. Stallmach, A.; Kesselmeier, M.; Bauer, M.; Gramlich, J.; Finke, K.; Fischer, A.; Fleischmann-Struzek, C.; Heutelbeck, A.; Katzer, K.; Mutschke, S.; Pletz, M. W.; Quickert, S.; Reinhart, K.; Stallmach, Z.; Walter, M.; Scherag, A.; Reuken, P. A., Comparison of fatigue, cognitive dysfunction and psychological disorders in post-COVID patients and patients after sepsis: is there a specific constellation? *Infection* **2022,** *50* (3), 661–669.
114. Mazza, M. G.; Palladini, M.; De Lorenzo, R.; Bravi, B.; Poletti, S.; Furlan, R.; Ciceri, F.; group, C.-B. O. C. S.; Rovere-Querini, P.; Benedetti, F., One-year mental health outcomes in a cohort of COVID-19 survivors. *J Psychiatr Res* **2021,** *145*, 118–124.
115. Poletti, S.; Palladini, M.; Mazza, M. G.; De Lorenzo, R.; group, C.-B. O. C. S.; Furlan, R.; Ciceri, F.; Rovere-Querini, P.; Benedetti, F., Long-term consequences of COVID-19 on cognitive functioning up to 6 months after discharge: role of depression and impact on quality of life. *Eur Arch Psychiatry Clin Neurosci* **2022,** *272* (5), 773–782.
116. Mechi, A.; Al-Khalidi, A.; Al-Darraji, R.; Al-Dujaili, M. N.; Al-Buthabhak, K.; Alareedh, M.; Shaghee, F.; Nafakhi, H., Long-term persistent symptoms of COVID-19 infection in patients with diabetes mellitus. *Int J Diabetes Dev Ctries* **2022,** *42* (1), 49–52.
117. Liu, T.; Wu, D.; Yan, W.; Wang, X.; Zhang, X.; Ma, K.; Chen, H.; Zeng, Z.; Qin, Y.; Wang, H.; Xing, M.; Xu, D.; Li, W.; Ni, M.; Zhu, L.; Chen, L.; Chen, G.; Qi, W.; Wu, T.; Yu, H.; Huang, J.; Han, M.; Zhu, W.; Guo, W.; Luo, X.; Chen, T.; Ning, Q., Twelve-Month Systemic Consequences of Coronavirus Disease 2019 (COVID-19) in Patients Discharged From Hospital: A Prospective Cohort Study in Wuhan, China. *Clin Infect Dis* **2022,** *74* (11), 1953–1965.
118. Klein, H.; Asseo, K.; Karni, N.; Benjamini, Y.; Nir-Paz, R.; Muszkat, M.; Israel, S.; Niv, M. Y., Onset, duration and unresolved symptoms, including smell and taste changes, in mild COVID-19 infection: a cohort study in Israeli patients. *Clin Microbiol Infect* **2021,** *27* (5), 769–74.
119. Savarraj, J. P. J.; Burkett, A. B.; Hinds, S. N.; Paz, A. S.; Assing, A.; Juneja, S.; Colpo, G. D.; Torres, L. F.; Cho, S. M.; Gusdon, A. M.; McCullough, L. D.; Choi, H. A., Pain and Other Neurological Symptoms Are Present at 3 Months After Hospitalization in COVID-19 Patients. *Front Pain Res (Lausanne)* **2021,** *2*, 737961.
120. Sakurada, Y.; Sunada, N.; Honda, H.; Tokumasu, K.; Otsuka, Y.; Nakano, Y.; Hanayama, Y.; Furukawa, M.; Hagiya, H.; Otsuka, F., Serial Changes of Long COVID Symptoms and Clinical Utility of Serum Antibody Titers for Evaluation of Long COVID. *J Clin Med* **2022,** *11* (5), 27.
121. Thyagaraj, V.; Rao, A.; Kulkarni, A.; Shankar, T.; R, N.; Unnikrishnan, H.; Kalaiah, K.; E, I.; Veluswamy, S. K.; Kumar, B. S. N.; Ravindra, S.; Shetty, N., Clinical and Laboratory Profile of Patients Visiting the Post-COVID-19 Clinic at a Tertiary Care Hospital: A Cross-Sectional Study. *Cureus* **2022,** *14* (3), e22888.
122. Gaur, R.; Asthana, S.; Yadav, R.; Ghuleliya, R.; Kumar, D.; Akhtar, M.; Gonnade, N.; Choudhary, A.; Mathew, M. M.; Gaur, N., Assessment of Physical Disability After Three Months in Patients Recovered From COVID-19: A Cross-Sectional Study. *Cureus* **2022,** *14* (1), e21618.
123. Yaksi, N.; Teker, A. G.; Imre, A., Long COVID in Hospitalized COVID-19 Patients: A Retrospective Cohort Study. *Iran J Public Health* **2022,** *51* (1), 88–95.
124. Huang, L.; Xu, X.; Zhang, L.; Zheng, D.; Liu, Y.; Feng, B.; Hu, J.; Lin, Q.; Xi, X.; Wang, Q.; Lin, M.; Zhou, X.; He, Z.; Weng, H.; Deng, Q.; Ding, B.; Guo, J.; Zhang, Z., Post-traumatic Stress Disorder Symptoms and Quality of Life of COVID-19 Survivors at 6-Month Follow-Up: A Cross-Sectional Observational Study. *Front Psychiatry* **2021,** *12*, 782478.
125. Fernandez-de-Las-Penas, C.; Martin-Guerrero, J. D.; Pellicer-Valero, O. J.; Navarro-Pardo, E.; Gomez-Mayordomo, V.; Cuadrado, M. L.; Arias-Navalon, J. A.; Cigaran-Mendez, M.; Hernandez-Barrera, V.; Arendt-Nielsen, L., Female Sex Is a Risk Factor Associated with Long-Term Post-COVID Related-Symptoms but Not with COVID-19 Symptoms: The LONG-COVID-EXP-CM Multicenter Study. *J Clin Med* **2022,** *11* (2), 14.
126. Vejen, M.; Hansen, E. F.; Al-Jarah, B. N. I.; Jensen, C.; Thaning, P.; Jeschke, K. N.; Ulrik, C. S., Hospital admission for COVID-19 pneumonitis - long-term impairment in quality of life and lung function. *Eur Clin Respir J* **2022,** *9* (1), 2024735.
127. Cacciatore, M.; Raggi, A.; Pilotto, A.; Cristillo, V.; Guastafierro, E.; Toppo, C.; Magnani, F. G.; Sattin, D.; Mariniello, A.; Silvaggi, F.; Cotti Piccinelli, S.; Zoppi, N.; Bonzi, G.; Gipponi, S.; Libri, I.; Bezzi, M.; Martelletti, P.; Leonardi, M.; Padovani, A., Neurological and Mental Health Symptoms Associated with Post-COVID-19 Disability in a Sample of Patients Discharged from a COVID-19 Ward: A Secondary Analysis. *Int J Environ Res Public Health* **2022,** *19* (7), 02.
128. Righi, E.; Mirandola, M.; Mazzaferri, F.; Dossi, G.; Razzaboni, E.; Zaffagnini, A.; Ivaldi, F.; Visentin, A.; Lambertenghi, L.; Arena, C.; Micheletto, C.; Gibellini, D.; Tacconelli, E., Determinants of persistence of symptoms and impact on physical and mental wellbeing in Long COVID: A prospective cohort study. *J Infect* **2022,** *84* (4), 566–572.
129. Knight, D. R. T.; Munipalli, B.; Logvinov, II; Halkar, M. G.; Mitri, G.; Dabrh, A. M. A.; Hines, S. L., Perception, Prevalence, and Prediction of Severe Infection and Post-acute Sequelae of COVID-19. *Am J Med Sci* **2022,** *363* (4), 295–304.

(*Continued*)

 © 2025. The Author(s).

Supplementary Table S1 (*Continued*)

130. Seessle, J.; Waterboer, T.; Hippchen, T.; Simon, J.; Kirchner, M.; Lim, A.; Muller, B.; Merle, U., Persistent Symptoms in Adult Patients 1 Year After Coronavirus Disease 2019 (COVID-19): A Prospective Cohort Study. *Clin Infect Dis* **2022,** *74* (7), 1191–1198.
131. Chen, X.; Li, Y.; Shao, T. R.; Yang, L. L.; Li, S. J.; Wang, X. J.; Li, A.; Wu, Y. Y.; Liu, X. F.; Liu, C. M.; Liu, Y. H.; Zeng, F.; Cen, Y., Some characteristics of clinical sequelae of COVID-19 survivors from Wuhan, China: A multi-center longitudinal study. *Influenza Other Respir Viruses* **2022,** *16* (3), 395–401.
132. Bai, F.; Tomasoni, D.; Falcinella, C.; Barbanotti, D.; Castoldi, R.; Mule, G.; Augello, M.; Mondatore, D.; Allegrini, M.; Cona, A.; Tesoro, D.; Tagliaferri, G.; Vigano, O.; Suardi, E.; Tincati, C.; Beringheli, T.; Varisco, B.; Battistini, C. L.; Piscopo, K.; Vegni, E.; Tavelli, A.; Terzoni, S.; Marchetti, G.; Monforte, A. D., Female gender is associated with long COVID syndrome: a prospective cohort study. *Clin Microbiol Infect* **2022,** *28* (4), 611 e9–611 e16.
133. Pilotto, A.; Cristillo, V.; Cotti Piccinelli, S.; Zoppi, N.; Bonzi, G.; Sattin, D.; Schiavolin, S.; Raggi, A.; Canale, A.; Gipponi, S.; Libri, I.; Frigerio, M.; Bezzi, M.; Leonardi, M.; Padovani, A., Long-term neurological manifestations of COVID-19: prevalence and predictive factors. *Neurol Sci* **2021,** *42* (12), 4903–4907.
134. Zangrillo, A.; Belletti, A.; Palumbo, D.; Calvi, M. R.; Guzzo, F.; Fominskiy, E. V.; Ortalda, A.; Nardelli, P.; Ripa, M.; Baiardo Redaelli, M.; Borghi, G.; Landoni, G.; D'Amico, F.; Marmiere, M.; Righetti, B.; Rocchi, M.; Saracino, M.; Tresoldi, M.; Dagna, L.; De Cobelli, F.; Group, C. O.-B. S., One-Year Multidisciplinary Follow-Up of Patients With COVID-19 Requiring Invasive Mechanical Ventilation. *J Cardiothorac Vasc Anesth* **2022,** *36* (5), 1354–1363.
135. Fang, X.; Ming, C.; Cen, Y.; Lin, H.; Zhan, K.; Yang, S.; Li, L.; Cao, G.; Li, Q.; Ma, X., Post-sequelae one year after hospital discharge among older COVID-19 patients: A multi-center prospective cohort study. *J Infect* **2022,** *84* (2), 179–186.
136. Kerget, B.; Celik, E.; Kerget, F.; Aksakal, A.; Ucar, E. Y.; Araz, O.; Akgun, M., Evaluation of 3-month follow-up of patients with postacute COVID-19 syndrome. *J Med Virol* **2022,** *94* (5), 2026–2034.
137. Mirfazeli, F. S.; Sarabi-Jamab, A.; Pereira-Sanchez, V.; Kordi, A.; Shariati, B.; Shariat, S. V.; Bahrami, S.; Nohesara, S.; Almasi-Dooghaee, M.; Faiz, S. H. R., Chronic fatigue syndrome and cognitive deficit are associated with acute-phase neuropsychiatric manifestations of COVID-19: A 9-month follow-up study. *Neurol Sci* **2022,** *43* (4), 2231–2239.
138. Banic, M.; Jankovic Makek, M.; Samarzija, M.; Mursic, D.; Boras, Z.; Trkes, V.; Baricevic, D.; Korsic, M.; Basara, L.; Jalusic Gluncic, T.; Vukic Dugac, A., Risk factors and severity of functional impairment in long COVID: a single-center experience in Croatia. *Croat Med J* **2022,** *63* (1), 27–35.
139. Mendelsohn, A. S.; Nath, N.; De Sa, A.; Von Pressentin, K. B., Two months follow-up of patients with non-critical COVID-19 in Cape Town, South Africa. *S Afr Fam Pract (2004)* **2022,** *64* (1), e1-e6.
140. Perez-Gonzalez, A.; Araujo-Ameijeiras, A.; Fernandez-Villar, A.; Crespo, M.; Poveda, E.; Cohort, C.-o. t. G. S. H. R. I., Long COVID in hospitalized and non-hospitalized patients in a large cohort in Northwest Spain, a prospective cohort study. *Sci Rep* **2022,** *12* (1), 3369.
141. Tejerina, F.; Catalan, P.; Rodriguez-Grande, C.; Adan, J.; Rodriguez-Gonzalez, C.; Munoz, P.; Aldamiz, T.; Diez, C.; Perez, L.; Fanciulli, C.; Garcia de Viedma, D.; Gregorio Maranon Microbiology, I. D. C. S. G., Post-COVID-19 syndrome. SARS-CoV-2 RNA detection in plasma, stool, and urine in patients with persistent symptoms after COVID-19. *BMC Infect Dis* **2022,** *22* (1), 211.
142. Bonifacio, L. P.; Csizmar, V. N. F.; Barbosa-Junior, F.; Pereira, A. P. S.; Koenigkam-Santos, M.; Wada, D. T.; Gaspar, G. G.; Carvalho, F. S.; Bollela, V. R.; Santana, R. C.; Souza, J. P.; Bellissimo-Rodrigues, F., Long-Term Symptoms among COVID-19 Survivors in Prospective Cohort Study, Brazil. *Emerg Infect Dis* **2022,** *28* (3), 730–733.
143. Bungenberg, J.; Humkamp, K.; Hohenfeld, C.; Rust, M. I.; Ermis, U.; Dreher, M.; Hartmann, N. K.; Marx, G.; Binkofski, F.; Finke, C.; Schulz, J. B.; Costa, A. S.; Reetz, K., Long COVID-19: Objectifying most self-reported neurological symptoms. *Ann Clin Transl Neurol* **2022,** *9* (2), 141–154.
144. Latronico, N.; Peli, E.; Calza, S.; Rodella, F.; Novelli, M. P.; Cella, A.; Marshall, J.; Needham, D. M.; Rasulo, F. A.; Piva, S.; Investigators, L., Physical, cognitive and mental health outcomes in 1-year survivors of COVID-19-associated ARDS. *Thorax* **2022,** *77* (3), 300–303.
145. Montenegro, P.; Moral, I.; Puy, A.; Cordero, E.; Chantada, N.; Cuixart, L.; Brotons, C., Prevalence of Post COVID-19 Condition in Primary Care: A Cross Sectional Study. *Int J Environ Res Public Health* **2022,** *19* (3), 06.
146. Karaarslan, F.; Guneri, F. D.; Kardes, S., Long COVID: rheumatologic/musculoskeletal symptoms in hospitalized COVID-19 survivors at 3 and 6 months. *Clin Rheumatol* **2022,** *41* (1), 289–296.
147. Titze-de-Almeida, R.; da Cunha, T. R.; Dos Santos Silva, L. D.; Ferreira, C. S.; Silva, C. P.; Ribeiro, A. P.; de Castro Moreira Santos Junior, A.; de Paula Brandao, P. R.; Silva, A. P. B.; da Rocha, M. C. O.; Xavier, M. E.; Titze-de-Almeida, S. S.; Shimizu, H. E.; Delgado-Rodrigues, R. N., Persistent, new-onset symptoms and mental health complaints in Long COVID in a Brazilian cohort of non-hospitalized patients. *BMC Infect Dis* **2022,** *22* (1), 133.
148. Fernandez-de-Las-Penas, C.; Palacios-Cena, D.; Gomez-Mayordomo, V.; Palacios-Cena, M.; Rodriguez-Jimenez, J.; de-la-Llave-Rincon, A. I.; Velasco-Arribas, M.; Fuensalida-Novo, S.; Ambite-Quesada, S.; Guijarro, C.; Cuadrado, M. L.; Florencio, L. L.; Arias-Navalon, J. A.; Ortega-Santiago, R.; Elvira-Martinez, C. M.; Molina-Trigueros, L. J.; Torres-Macho, J.; Sebastian-Viana, T.; Canto-Diez, M. G.; Cigaran-Mendez, M.; Hernandez-Barrera, V.; Arendt-Nielsen, L., Fatigue and Dyspnoea as Main Persistent Post-COVID-19 Symptoms in Previously Hospitalized Patients: Related Functional Limitations and Disability. *Respiration* **2022,** *101* (2), 132–141.
149. Kim, Y.; Bitna, H.; Kim, S. W.; Chang, H. H.; Kwon, K. T.; Bae, S.; Hwang, S., Post-acute COVID-19 syndrome in patients after 12 months from COVID-19 infection in Korea. *BMC Infect Dis* **2022,** *22* (1), 93.
150. Noviello, D.; Costantino, A.; Muscatello, A.; Bandera, A.; Consonni, D.; Vecchi, M.; Basilisco, G., Functional gastrointestinal and somatoform symptoms five months after SARS-CoV-2 infection: A controlled cohort study. *Neurogastroenterol Motil* **2022,** *34* (2), e14187.
151. Tleyjeh, I. M.; Saddik, B.; Ramakrishnan, R. K.; AlSwaidan, N.; AlAnazi, A.; Alhazmi, D.; Aloufi, A.; AlSumait, F.; Berbari, E. F.; Halwani, R., Long term predictors of breathlessness, exercise intolerance, chronic fatigue and well-being in hospitalized patients with COVID-19: A cohort study with 4 months median follow-up. *J Infect Public Health* **2022,** *15* (1), 21–28.
152. Fernandez-de-Las-Penas, C.; Torres-Macho, J.; Elvira-Martinez, C. M.; Molina-Trigueros, L. J.; Sebastian-Viana, T.; Hernandez-Barrera, V., Obesity is associated with a greater number of long-term post-COVID symptoms and poor sleep quality: A multicentre case-control study. *Int J Clin Pract* **2021,** *75* (12), e14917.

Supplementary Table S1 (*Continued*)

153. Asadi-Pooya, A. A.; Akbari, A.; Emami, A.; Lotfi, M.; Rostamihosseinkhani, M.; Nemati, H.; Barzegar, Z.; Kabiri, M.; Zeraatpisheh, Z.; Farjoud-Kouhanjani, M.; Jafari, A.; Sasannia, F.; Ashrafi, S.; Nazeri, M.; Nasiri, S.; Shahisavandi, M., Risk Factors Associated with Long COVID Syndrome: A Retrospective Study. *Iran J Med Sci* **2021,** *46* (6), 428–436.
154. Tessitore, E.; Handgraaf, S.; Poncet, A.; Achard, M.; Hofer, S.; Carballo, S.; Marti, C.; Follonier, C.; Girardin, F.; Mach, F.; Carballo, D., Symptoms and quality of life at 1-year follow up of patients discharged after an acute COVID-19 episode. *Swiss Med Wkly* **2021,** *151*, w30093.
155. Hossain, M. A.; Hossain, K. M. A.; Saunders, K.; Uddin, Z.; Walton, L. M.; Raigangar, V.; Sakel, M.; Shafin, R.; Hossain, M. S.; Kabir, M. F.; Faruqui, R.; Rana, M. S.; Ahmed, M. S.; Chakrovorty, S. K.; Hossain, M. A.; Jahid, I. K., Prevalence of Long COVID symptoms in Bangladesh: a prospective Inception Cohort Study of COVID-19 survivors. *BMJ Glob Health* **2021,** *6* (12), 12.
156. Tleyjeh, I. M.; Saddik, B.; AlSwaidan, N.; AlAnazi, A.; Ramakrishnan, R. K.; Alhazmi, D.; Aloufi, A.; AlSumait, F.; Berbari, E.; Halwani, R., Prevalence and predictors of Post-Acute COVID-19 Syndrome (PACS) after hospital discharge: A cohort study with 4 months median follow-up. *PLoS ONE* **2021,** *16* (12), e0260568.
157. Tan, S.; Hewitt, L.; Cuenca, J.; Risi, D., Outcomes of COVID-19 in the community: A prospective cohort study. *Aust J Gen Pract* **2021,** *50* (12), 922–928.
158. Zhao, Y.; Yang, C.; An, X.; Xiong, Y.; Shang, Y.; He, J.; Qiu, Y.; Zhang, N.; Huang, L.; Jia, J.; Xu, Q.; Zhang, L.; Zhao, J.; Pei, G.; Luo, H.; Wang, J.; Li, Q.; Gao, Y.; Xu, A., Follow-up study on COVID-19 survivors one year after discharge from hospital. *Int J Infect Dis* **2021,** *112*, 173–182.
159. Zhou, F.; Tao, M.; Shang, L.; Liu, Y.; Pan, G.; Jin, Y.; Wang, L.; Hu, S.; Li, J.; Zhang, M.; Fu, Y.; Yang, S., Assessment of Sequelae of COVID-19 Nearly 1 Year After Diagnosis. *Front Med (Lausanne)* **2021,** *8*, 717194.
160. Bottemanne, H.; Gouraud, C.; Hulot, J. S.; Blanchard, A.; Ranque, B.; Lahlou-Laforet, K.; Limosin, F.; Gunther, S.; Lebeaux, D.; Lemogne, C., Do Anxiety and Depression Predict Persistent Physical Symptoms After a Severe COVID-19 Episode? A Prospective Study. *Front Psychiatry* **2021,** *12*, 757685.
161. Molhave, M.; Leth, S.; Gunst, J. D.; Jensen-Fangel, S.; Ostergaard, L.; Wejse, C.; Agergaard, J., Long-Term Symptoms among Hospitalized COVID-19 Patients 48 Weeks after Discharge-A Prospective Cohort Study. *J Clin Med* **2021,** *10* (22), 15.
162. Boesl, F.; Audebert, H.; Endres, M.; Pruss, H.; Franke, C., A Neurological Outpatient Clinic for Patients With Post-COVID-19 Syndrome - A Report on the Clinical Presentations of the First 100 Patients. *Front Neurol* **2021,** *12*, 738405.
163. Dini, M.; Poletti, B.; Tagini, S.; Reitano, M. R.; Allocco, E.; Mazzocco, K.; Pravettoni, G.; Dell'Osso, B.; Monforte, A. D.; Centanni, S.; Priori, A.; Ferrucci, R., Resilience, Psychological Well-Being and Daily Functioning Following Hospitalization for Respiratory Distress Due to SARS-CoV-2 Infection. *Healthcare (Basel)* **2021,** *9* (9), 04.
164. Lamontagne, S. J.; Winters, M. F.; Pizzagalli, D. A.; Olmstead, M. C., Post-acute sequelae of COVID-19: Evidence of mood & cognitive impairment. *Brain Behav Immun Health* **2021,** *17*, 100347.
165. Rao, S.; Amara, V.; Chaudhuri, S.; Rao, B. K.; Todur, P., "Post-COVID-19 syndrome": The New Pandemic Affecting Healthcare Workers and How the Frontline Warriors Are Battling it. *Indian J Palliat Care* **2021,** *27* (2), 313–318.
166. Zayet, S.; Zahra, H.; Royer, P. Y.; Tipirdamaz, C.; Mercier, J.; Gendrin, V.; Lepiller, Q.; Marty-Quinternet, S.; Osman, M.; Belfeki, N.; Toko, L.; Garnier, P.; Pierron, A.; Plantin, J.; Messin, L.; Villemain, M.; Bouiller, K.; Klopfenstein, T., Post-COVID-19 Syndrome: Nine Months after SARS-CoV-2 Infection in a Cohort of 354 Patients: Data from the First Wave of COVID-19 in Nord Franche-Comte Hospital, France. *Microorganisms* **2021,** *9* (8), 12.
167. Kim, Y.; Kim, S. W.; Chang, H. H.; Kwon, K. T.; Bae, S.; Hwang, S., Significance and Associated Factors of Long-Term Sequelae in Patients after Acute COVID-19 Infection in Korea. *Infect Chemother* **2021,** *53* (3), 463–476.
168. Lombardo, M. D. M.; Foppiani, A.; Peretti, G. M.; Mangiavini, L.; Battezzati, A.; Bertoli, S.; Martinelli Boneschi, F.; Zuccotti, G. V., Long-Term Coronavirus Disease 2019 Complications in Inpatients and Outpatients: A One-Year Follow-up Cohort Study. *Open Forum Infect Dis* **2021,** *8* (8), ofab384.
169. Castro, V. M.; Rosand, J.; Giacino, J. T.; McCoy, T. H.; Perlis, R. H., Case-control study of neuropsychiatric symptoms following COVID-19 hospitalization in 2 academic health systems. *medRxiv* **2021,** *14*, 14.
170. Bastola, A.; Nepal, R.; Shrestha, B.; Maharjan, K.; Shrestha, S.; Chalise, B. S.; Neupane, J., Persistent Symptoms in Post-COVID-19 Patients Attending Follow-Up OPD at Sukraraj Tropical and Infectious Disease Hospital (STIDH), Kathmandu, Nepal. *Trop Med Infect Dis* **2021,** *6* (3), 28.
171. Gramaglia, C.; Gambaro, E.; Bellan, M.; Balbo, P. E.; Baricich, A.; Sainaghi, P. P.; Pirisi, M.; Baldon, G.; Battistini, S.; Binda, V.; Feggi, A.; Gai, M.; Gattoni, E.; Jona, A.; Lorenzini, L.; Marangon, D.; Martelli, M.; Prosperini, P.; Zeppegno, P.; Group, N.-M. C., Mid-term Psychiatric Outcomes of Patients Recovered From COVID-19 From an Italian Cohort of Hospitalized Patients. *Front Psychiatry* **2021,** *12*, 667385.
172. Righi, E.; Mirandola, M.; Mazzaferri, F.; Razzaboni, E.; Zaffagnini, A.; Erbogasto, A.; Vecchia, I. D.; Auerbach, N.; Ivaldi, F.; Mongardi, M.; Minuz, P.; Milella, M.; Mehrabi, S.; Olivieri, O.; Girelli, D.; Polati, E.; Micheletto, C.; Tacconelli, E., Long-Term Patient-Centred Follow-up in a Prospective Cohort of Patients with COVID-19. *Infect Dis Ther* **2021,** *10* (3), 1579–1590.
173. Augustin, M.; Schommers, P.; Stecher, M.; Dewald, F.; Gieselmann, L.; Gruell, H.; Horn, C.; Vanshylla, K.; Cristanziano, V. D.; Osebold, L.; Roventa, M.; Riaz, T.; Tschernoster, N.; Altmueller, J.; Rose, L.; Salomon, S.; Priesner, V.; Luers, J. C.; Albus, C.; Rosenkranz, S.; Gathof, B.; Fatkenheuer, G.; Hallek, M.; Klein, F.; Suarez, I.; Lehmann, C., Post-COVID syndrome in non-hospitalised patients with COVID-19: a longitudinal prospective cohort study. *Lancet Reg Health Eur* **2021,** *6*, 100122.
174. Leth, S.; Gunst, J. D.; Mathiasen, V.; Hansen, K.; Sogaard, O.; Ostergaard, L.; Jensen-Fangel, S.; Storgaard, M.; Agergaard, J., Persistent Symptoms in Patients Recovering From COVID-19 in Denmark. *Open Forum Infect Dis* **2021,** *8* (4), ofab042.
175. Miyazato, Y.; Morioka, S.; Tsuzuki, S.; Akashi, M.; Osanai, Y.; Tanaka, K.; Terada, M.; Suzuki, M.; Kutsuna, S.; Saito, S.; Hayakawa, K.; Ohmagari, N., Prolonged and Late-Onset Symptoms of Coronavirus Disease 2019. *Open Forum Infect Dis* **2020,** *7* (11), ofaa507.
176. Heightman, M.; Prashar, J.; Hillman, T. E.; Marks, M.; Livingston, R.; Ridsdale, H. A.; Bell, R.; Zandi, M.; McNamara, P.; Chauhan, A.; Denneny, E.; Astin, R.; Purcell, H.; Attree, E.; Hishmeh, L.; Prescott, G.; Evans, R.; Mehta, P.; Brennan, E.; Brown, J. S.; Porter, J.; Logan, S.; Wall, E.; Dehbi, H. M.; Cone, S.; Banerjee, A., Post-COVID-19 assessment in a specialist clinical service: a 12-month, single-center, prospective study in 1325 individuals. *BMJ Open Respir Res* **2021,** *8* (1), 11.

(*Continued*)

Supplementary Table S1 (*Continued*)

177. Munblit, D.; Bobkova, P.; Spiridonova, E.; Shikhaleva, A.; Gamirova, A.; Blyuss, O.; Nekliudov, N.; Bugaeva, P.; Andreeva, M.; DunnGalvin, A.; Comberiati, P.; Apfelbacher, C.; Genuneit, J.; Avdeev, S.; Kapustina, V.; Guekht, A.; Fomin, V.; Svistunov, A. A.; Timashev, P.; Subbot, V. S.; Royuk, V. V.; Drake, T. M.; Hanson, S. W.; Merson, L.; Carson, G.; Horby, P.; Sigfrid, L.; Scott, J. T.; Semple, M. G.; Warner, J. O.; Vos, T.; Olliaro, P.; Glybochko, P.; Butnaru, D.; Sechenov Stop, C. R. T., Incidence and risk factors for persistent symptoms in adults previously hospitalized for COVID-19. *Clin Exp Allergy* **2021,** *51* (9), 1107–1120.
178. Nehme, M.; Braillard, O.; Chappuis, F.; Courvoisier, D. S.; Guessous, I.; CoviCare Study, T., Prevalence of Symptoms More Than Seven Months After Diagnosis of Symptomatic COVID-19 in an Outpatient Setting. *Ann Intern Med* **2021,** *174* (9), 1252–1260.
179. Naik, S.; Haldar, S. N.; Soneja, M.; Mundadan, N. G.; Garg, P.; Mittal, A.; Desai, D.; Trilangi, P. K.; Chakraborty, S.; Begam, N. N.; Bhattacharya, B.; Maher, G.; Mahishi, N.; Rajanna, C.; Kumar, S. S.; Arunan, B.; Kirtana, J.; Gupta, A.; Patidar, D.; Kodan, P.; Sethi, P.; Ray, A.; Jorwal, P.; Kumar, A.; Nischal, N.; Sinha, S.; Biswas, A.; Wig, N., Post COVID-19 sequelae: A prospective observational study from Northern India. *Drug Discov Ther* **2021,** *15* (5), 254–260.
180. Kerr, C.; S, O. R.; Creagh, D.; Hughes, G.; Geary, U.; Colgan, M. P.; Canning, C.; Martin, Z.; Merry, C.; Noonan, N.; Bergin, C., Acceptability of and symptom findings from an online symptom check-in tool for COVID-19 outpatient follow-up among a predominantly healthcare worker population. *BMJ Open* **2021,** *11* (9), e050444.
181. Zhang, X.; Wang, F.; Shen, Y.; Zhang, X.; Cen, Y.; Wang, B.; Zhao, S.; Zhou, Y.; Hu, B.; Wang, M.; Liu, Y.; Miao, H.; Jones, P.; Ma, X.; He, Y.; Cao, G.; Cheng, L.; Li, L., Symptoms and Health Outcomes Among Survivors of COVID-19 Infection 1 Year After Discharge From Hospitals in Wuhan, China. *JAMA Netw Open* **2021,** *4* (9), e2127403.
182. Tiwari, B.; Ghimire, M.; Bhatta, G.; Banstola, H.; Tiwari, B.; Twayana, A.; Shrestha, K., Persistent Symptoms in Non-critical COVID-19 Patients at Two Months Follow-Up in a District Hospital: A Descriptive Cross-sectional Study. *JNMA J Nepal Med Assoc* **2021,** *59* (238), 550–553.
183. Wanga, V.; Chevinsky, J. R.; Dimitrov, L. V.; Gerdes, M. E.; Whitfield, G. P.; Bonacci, R. A.; Nji, M. A. M.; Hernandez-Romieu, A. C.; Rogers-Brown, J. S.; McLeod, T.; Rushmore, J.; Lutfy, C.; Bushman, D.; Koumans, E.; Saydah, S.; Goodman, A. B.; Coleman King, S. M.; Jackson, B. R.; Cope, J. R., Long-Term Symptoms Among Adults Tested for SARS-CoV-2 - United States, January 2020-April 2021. *MMWR Morb Mortal Wkly Rep* **2021,** *70* (36), 1235–1241.
184. Van Herck, M.; Goertz, Y. M. J.; Houben-Wilke, S.; Machado, F. V. C.; Meys, R.; Delbressine, J. M.; Vaes, A. W.; Burtin, C.; Posthuma, R.; Franssen, F. M. E.; Hajian, B.; Vijlbrief, H.; Spies, Y.; van 't Hul, A. J.; Janssen, D. J. A.; Spruit, M. A., Severe Fatigue in Long COVID: Web-Based Quantitative Follow-up Study in Members of Online Long COVID Support Groups. *J Med Internet Res* **2021,** *23* (9), e30274.
185. Grover, S.; Sahoo, S.; Mishra, E.; Gill, K. S.; Mehra, A.; Nehra, R.; Suman, A.; Bhalla, A.; Puri, G. D., Fatigue, perceived stigma, self-reported cognitive deficits and psychological morbidity in patients recovered from COVID-19 infection. *Asian J Psychiatr* **2021,** *64*, 102815.
186. Huang, L.; Yao, Q.; Gu, X.; Wang, Q.; Ren, L.; Wang, Y.; Hu, P.; Guo, L.; Liu, M.; Xu, J.; Zhang, X.; Qu, Y.; Fan, Y.; Li, X.; Li, C.; Yu, T.; Xia, J.; Wei, M.; Chen, L.; Li, Y.; Xiao, F.; Liu, D.; Wang, J.; Wang, X.; Cao, B., 1-year outcomes in hospital survivors with COVID-19: a longitudinal cohort study. *Lancet* **2021,** *398* (10302), 747–758.
187. Soraas, A.; Kalleberg, K. T.; Dahl, J. A.; Soraas, C. L.; Myklebust, T. A.; Axelsen, E.; Lind, A.; Baevre-Jensen, R.; Jorgensen, S. B.; Istre, M. S.; Kjetland, E. F.; Ursin, G., Persisting symptoms three to eight months after non-hospitalized COVID-19, a prospective cohort study. *PLoS ONE* **2021,** *16* (8), e0256142.
188. Bakilan, F.; Gokmen, I. G.; Ortanca, B.; Ucan, A.; Eker Guvenc, S.; Sahin Mutlu, F.; Gokmen, H. M.; Ekim, A., Musculoskeletal symptoms and related factors in postacute COVID-19 patients. *Int J Clin Pract* **2021,** *75* (11), e14734.
189. Bell, M. L.; Catalfamo, C. J.; Farland, L. V.; Ernst, K. C.; Jacobs, E. T.; Klimentidis, Y. C.; Jehn, M.; Pogreba-Brown, K., Post-acute sequelae of COVID-19 in a non-hospitalized cohort: Results from the Arizona CoVHORT. *PLoS ONE* **2021,** *16* (8), e0254347.
190. Kanberg, N.; Simren, J.; Eden, A.; Andersson, L. M.; Nilsson, S.; Ashton, N. J.; Sundvall, P. D.; Nellgard, B.; Blennow, K.; Zetterberg, H.; Gisslen, M., Neurochemical signs of astrocytic and neuronal injury in acute COVID-19 normalizes during long-term follow-up. *EBioMedicine* **2021,** *70*, 103512.
191. Kayaaslan, B.; Eser, F.; Kalem, A. K.; Kaya, G.; Kaplan, B.; Kacar, D.; Hasanoglu, I.; Coskun, B.; Guner, R., Post-COVID syndrome: A single-center questionnaire study on 1007 participants recovered from COVID-19. *J Med Virol* **2021,** *93* (12), 6566–6574.
192. Menges, D.; Ballouz, T.; Anagnostopoulos, A.; Aschmann, H. E.; Domenghino, A.; Fehr, J. S.; Puhan, M. A., Burden of post-COVID-19 syndrome and implications for healthcare service planning: A population-based cohort study. *PLoS ONE* **2021,** *16* (7), e0254523.
193. Bierle, D. M.; Aakre, C. A.; Grach, S. L.; Salonen, B. R.; Croghan, I. T.; Hurt, R. T.; Ganesh, R., Central Sensitization Phenotypes in Post Acute Sequelae of SARS-CoV-2 Infection (PASC): Defining the Post COVID Syndrome. *J Prim Care Community Health* **2021,** *12*, 21501327211030826.
194. Ganesh, R.; Ghosh, A. K.; Nyman, M. A.; Croghan, I. T.; Grach, S. L.; Anstine, C. V.; Salonen, B. R.; Hurt, R. T., PROMIS Scales for Assessment of Persistent Post-COVID Symptoms: A Cross Sectional Study. *J Prim Care Community Health* **2021,** *12*, 21501327211030413.
195. Blomberg, B.; Mohn, K. G.; Brokstad, K. A.; Zhou, F.; Linchausen, D. W.; Hansen, B. A.; Lartey, S.; Onyango, T. B.; Kuwelker, K.; Saevik, M.; Bartsch, H.; Tondel, C.; Kittang, B. R.; Bergen, C.-R. G.; Cox, R. J.; Langeland, N., Long COVID in a prospective cohort of home-isolated patients. *Nat Med* **2021,** *27* (9), 1607–1613.
196. Bliddal, S.; Banasik, K.; Pedersen, O. B.; Nissen, J.; Cantwell, L.; Schwinn, M.; Tulstrup, M.; Westergaard, D.; Ullum, H.; Brunak, S.; Tommerup, N.; Feenstra, B.; Geller, F.; Ostrowski, S. R.; Gronbaek, K.; Nielsen, C. H.; Nielsen, S. D.; Feldt-Rasmussen, U., Acute and persistent symptoms in non-hospitalized PCR-confirmed COVID-19 patients. *Sci Rep* **2021,** *11* (1), 13153.
197. Ghosn, J.; Piroth, L.; Epaulard, O.; Le Turnier, P.; Mentre, F.; Bachelet, D.; Laouenan, C.; French, C. c. s.; investigators, g., Persistent COVID-19 symptoms are highly prevalent 6 months after hospitalization: results from a large prospective cohort. *Clin Microbiol Infect* **2021,** *27* (7), 1041 e1–1041 e4.
198. Labarca, G.; Henriquez-Beltran, M.; Lastra, J.; Enos, D.; Llerena, F.; Cigarroa, I.; Lamperti, L.; Ormazabal, V.; Ramirez, C.; Espejo, E.; Canales, N.; Fuentes, F.; Horta, G.; Fernandez-Bussy, S.; Nova-Lamperti, E., Analysis of clinical symptoms, radiological changes and pulmonary function data 4 months after COVID-19. *Clin Respir J* **2021,** *15* (9), 992–1002.

 © 2025. The Author(s).

Supplementary Table S1 (*Continued*)

199. Albu, S.; Zozaya, N. R.; Murillo, N.; Garcia-Molina, A.; Chacon, C. A. F.; Kumru, H., What's going on following acute covid-19? Clinical characteristics of patients in an out-patient rehabilitation program. *NeuroRehabilitation* **2021,** *48* (4), 469–480.
200. Fernandez-de-Las-Penas, C.; Guijarro, C.; Plaza-Canteli, S.; Hernandez-Barrera, V.; Torres-Macho, J., Prevalence of Post-COVID-19 Cough One Year After SARS-CoV-2 Infection: A Multicenter Study. *Lung* **2021,** *199* (3), 249–253.
201. Mattioli, F.; Stampatori, C.; Righetti, F.; Sala, E.; Tomasi, C.; De Palma, G., Neurological and cognitive sequelae of Covid-19: a four month follow-up. *J Neurol* **2021,** *268* (12), 4422–4428.
202. Shang, Y. F.; Liu, T.; Yu, J. N.; Xu, X. R.; Zahid, K. R.; Wei, Y. C.; Wang, X. H.; Zhou, F. L., Half-year follow-up of patients recovering from severe COVID-19: Analysis of symptoms and their risk factors. *J Intern Med* **2021,** *290* (2), 444–450.
203. Fernandez-de-Las-Penas, C.; Rodriguez-Jimenez, J.; Fuensalida-Novo, S.; Palacios-Cena, M.; Gomez-Mayordomo, V.; Florencio, L. L.; Hernandez-Barrera, V.; Arendt-Nielsen, L., Myalgia as a symptom at hospital admission by severe acute respiratory syndrome coronavirus 2 infection is associated with persistent musculoskeletal pain as long-term post-COVID sequelae: a case-control study. *Pain* **2021,** *162* (12), 2832–2840.
204. Mahmud, R.; Rahman, M. M.; Rassel, M. A.; Monayem, F. B.; Sayeed, S.; Islam, M. S.; Islam, M. M., Post-COVID-19 syndrome among symptomatic COVID-19 patients: A prospective cohort study in a tertiary care center of Bangladesh. *PLoS ONE* **2021,** *16* (4), e0249644.
205. Osikomaiya, B.; Erinoso, O.; Wright, K. O.; Odusola, A. O.; Thomas, B.; Adeyemi, O.; Bowale, A.; Adejumo, O.; Falana, A.; Abdus-Salam, I.; Ogboye, O.; Osibogun, A.; Abayomi, A., 'Long COVID': persistent COVID-19 symptoms in survivors managed in Lagos State, Nigeria. *BMC Infect Dis* **2021,** *21* (1), 304.
206. Stavem, K.; Ghanima, W.; Olsen, M. K.; Gilboe, H. M.; Einvik, G., Prevalence and Determinants of Fatigue after COVID-19 in Non-Hospitalized Subjects: A Population-Based Study. *Int J Environ Res Public Health* **2021,** *18* (4), 19.
207. Trinkmann, F.; Muller, M.; Reif, A.; Kahn, N.; Kreuter, M.; Trudzinski, F.; Eichinger, M.; Heussel, C. P.; Herth, F. J. F.; Lung Network, R.-N.-R., Residual symptoms and lower lung function in patients recovering from SARS-CoV-2 infection. *Eur Respir J* **2021,** *57* (2), 02.
208. Huang, C.; Huang, L.; Wang, Y.; Li, X.; Ren, L.; Gu, X.; Kang, L.; Guo, L.; Liu, M.; Zhou, X.; Luo, J.; Huang, Z.; Tu, S.; Zhao, Y.; Chen, L.; Xu, D.; Li, Y.; Li, C.; Peng, L.; Li, Y.; Xie, W.; Cui, D.; Shang, L.; Fan, G.; Xu, J.; Wang, G.; Wang, Y.; Zhong, J.; Wang, C.; Wang, J.; Zhang, D.; Cao, B., 6-month consequences of COVID-19 in patients discharged from hospital: a cohort study. *Lancet* **2021,** *397* (10270), 220–232.
209. Vedel Sørensen, A. I.; Spiliopoulos, L.; Bager, P.; Nielsen, N. M.; Hansen, J. V.; Koch, A.; Meder, I. K.; Ethelberg, S.; Hviid, A., Post-acute symptoms, new onset diagnoses and health problems 6 to 12 months after SARS-CoV-2 infection: a nationwide questionnaire study in the adult Danish population. *medRxiv.* **2022,** *28*.
210. Arjun, M. C.; Singh, A. K.; Pal, D.; Das, K.; Gajjala, A.; Venkateshan, M.; Mishra, B.; Patro, B. K.; Mohapatra, P. R.; Subba, S. H., Prevalence, characteristics, and predictors of Long COVID among diagnosed cases of COVID-19. *medRxiv.* **2022,** *08*.
211. Wose Kinge, C. N.; Hanekom, S.; Smith, A. L.; Akpan, F.; Mothibi, E.; Maotoe, T.; Lebatie, F.; Majuba, P.; Sanne, I.; Chasela, C., Persistent Symptoms Among Frontline Health Workers Post-acute COVID-19 Infection. *medRxiv.* **2021,** *05*.
212. Silva, L. S.; Joao, R. B.; Nogueira, M. H.; Aventurato, I. K.; de Campos, B. M.; de Brito, M. R.; Alvim, M. K. M.; Ludwig, G. V. N.; Rocha, C.; Souza, T. K. A. S.; da Costa, B. A.; Mendes, M. J.; Waku, T.; de Oliveira Boldrini, V.; Brunetti, N. S.; Baptista, S. N.; Schmitt, G. d. S.; de Sousa, J. G. D.; de Oliveira Cardoso, T. A. M.; Vieira, A. S.; Santos, L. M. B.; Farias, A. d. S.; Cendes, F.; Yasuda, C. L., Functional and microstructural brain abnormalities, fatigue, and cognitive dysfunction after mild COVID-19. *medRxiv.* **2021,** *24*.
213. Hassan, H. N.; Altabatbaee, K. S. N., Post Covid-19 Syndrome: A Cross Sectional Study in Baghdad. *Medico-legal Update* **2022,** *22* (2), 50–57.
214. Margalit, I.; Yelin, D.; Sagi, M.; Rahat, M. M.; Sheena, L.; Mizrahi, N.; Gordin, Y.; Agmon, H.; Epstein, N. K.; Atamna, A.; Tishler, O.; Daitch, V.; Babich, T.; Abecasis, D.; Yarom, Y.; Kazum, S.; Shitenberg, D.; Baltaxe, E.; Elkana, O.; Shapira-Lichter, I.; Leibovici, L.; Yahav, D., Risk Factors and Multidimensional Assessment of Long Coronavirus Disease Fatigue: A Nested Case-Control Study. *Clin Infect Dis* **2022,** *75* (10), 1688–1697.
215. Kenny, G.; McCann, K.; O'Brien, C.; Savinelli, S.; Tinago, W.; Yousif, O.; Lambert, J. S.; O'Broin, C.; Feeney, E. R.; De Barra, E.; Doran, P.; Mallon, P. W. G.; All-Ireland Infectious Diseases Cohort Study, G., Identification of Distinct Long COVID Clinical Phenotypes Through Cluster Analysis of Self-Reported Symptoms. *Open Forum Infect Dis* **2022,** *9* (4), ofac060.
216. Akova, I., Determination of Ongoing Symptoms, Quality of Life Levels, and Risk Factors in Post-COVID-19 Patients. *Erciyes Medical Journal* **2021,** *44(2)*, 208–215.
217. D'Souza M, M.; Kaushik, A.; Dsouza, J. M.; Kanwar, R.; Lodhi, V.; Sharma, R.; Mishra, A. K., Does the initial chest radiograph severity in COVID-19 impact the short- and long-term outcome? - a perspective from India. *Infect Dis (Lond)* **2022,** *54* (5), 335–344.
218. Banu, N.; Algosaibi, A. A.; Alhazza, K. A.; Al Janbi, L. M.; Daud Ali, M.; Ahmad, A., Prevalence of Long-term Post COVID-19 Symptom and Blood Group Correlation. *Journal of Young Pharmacists* **2021,** *13* (4), 400–404.
219. Duggal, P.; Penson, T.; Manley, H. N.; Vergara, C.; Munday, R. M.; Duchen, D.; Linton, E. A.; Zurn, A.; Keruly, J. C.; Mehta, S. H.; Thomas, D. L., Post-sequelae symptoms and comorbidities after COVID-19. *J Med Virol* **2022,** *94* (5), 2060–2066.
220. Rivera-Izquierdo, M.; Lainez-Ramos-Bossini, A. J.; de Alba, I. G.; Ortiz-Gonzalez-Serna, R.; Serrano-Ortiz, A.; Fernandez-Martinez, N. F.; Ruiz-Montero, R.; Cervilla, J. A., Long COVID 12 months after discharge: persistent symptoms in patients hospitalised due to COVID-19 and patients hospitalised due to other causes-a multicentre cohort study. *BMC Med* **2022,** *20* (1), 92.
221. Elkan, M.; Dvir, A.; Zaidenstein, R.; Keller, M.; Kagansky, D.; Hochman, C.; Koren, R., Patient-Reported Outcome Measures After Hospitalization During the COVID-19 Pandemic: A Survey Among COVID-19 and Non-COVID-19 Patients. *Int J Gen Med* **2021,** *14*, 4829–4836.
222. Pela, G.; Goldoni, M.; Cavalli, C.; Perrino, F.; Tagliaferri, S.; Frizzelli, A.; Mori, P. A.; Majori, M.; Aiello, M.; Sverzellati, N.; Corradi, M.; Chetta, A., Long-Term Cardiac Sequelae in Patients Referred into a Diagnostic Post-COVID-19 Pathway: The Different Impacts on the Right and Left Ventricles. *Diagnostics (Basel)* **2021,** *11* (11).
223. Ram-Mohan, N.; Kim, D.; Rogers, A. J.; Blish, C. A.; Nadeau, K. C.; Blomkalns, A. L.; Yang, S., Association Between SARS-CoV-2 RNAemia and Postacute Sequelae of COVID-19. *Open Forum Infect Dis* **2022,** *9* (2), ofab646.

(*Continued*)

Supplementary Table S1 (*Continued*)

224. Romero-Duarte, A.; Rivera-Izquierdo, M.; Lainez-Ramos-bossini, A. J.; Redruello-Guerrero, P.; Cardenas-Cruz, A., Factors associated with readmission to the Emergency Department in a cohort of COVID-19 hospitalized patients. *Signa Vitae* **2021,** *18(1)*, 47–54.
225. Mendez, R.; Balanza-Martinez, V.; Luperdi, S. C.; Estrada, I.; Latorre, A.; Gonzalez-Jimenez, P.; Bouzas, L.; Yepez, K.; Ferrando, A.; Reyes, S.; Menendez, R., Long-term neuropsychiatric outcomes in COVID-19 survivors: A 1-year longitudinal study. *J Intern Med* **2022,** *291* (2), 247–251.
226. Hema, H. A.; Jayaram, S.; Ravi, S.; Lakshmeesha, T., A Study on Persistent Symptoms in Patients Who Were Discharged from a Dedicated Covid Hospital Intensive Care Unit (Icu). *European Journal of Molecular and Clinical Medicine* **2021,** *8(4)*, 2034–2039.
227. Sampaio Rocha-Filho, P. A.; Albuquerque, P. M.; Carvalho, L.; Dandara Pereira Gama, M.; Magalhaes, J. E., Headache, anosmia, ageusia and other neurological symptoms in COVID-19: a cross-sectional study. *J Headache Pain* **2022,** *23* (1), 2.
228. Wong-Chew, R. M.; Rodriguez Cabrera, E. X.; Rodriguez Valdez, C. A.; Lomelin-Gascon, J.; Morales-Juarez, L.; de la Cerda, M. L. R.; Villa-Romero, A. R.; Arce Fernandez, S.; Serratos Fernandez, M.; Bello, H. H.; Castaneda, L. M.; Avendano, M. A.; Hernandez-Cruz, J. A.; Alvarez Martinez, N.; Fernanda Contreras, L.; Rafael Gonzalez de la Cerda, L.; Juarez Flores, A.; Martinez-Juarez, L. A.; Alvarez-Hernandez, D. A.; Gallardo-Rincon, H.; Fajardo Dolci, G.; Tapia-Conyer, R.; Valdez-Vazquez, R. R., Symptom cluster analysis of long COVID-19 in patients discharged from the Temporary COVID-19 Hospital in Mexico City. *Ther Adv Infect Dis* **2022,** *9* (no pagination), 20499361211069264.
229. Peghin, M.; Palese, A.; Venturini, M.; De Martino, M.; Gerussi, V.; Graziano, E.; Bontempo, G.; Marrella, F.; Tommasini, A.; Fabris, M.; Curcio, F.; Isola, M.; Tascini, C., Post-COVID-19 symptoms 6 months after acute infection among hospitalized and non-hospitalized patients. *Clin Microbiol Infect* **2021,** *27* (10), 1507–1513.
230. Zhou, M.; Cai, J.; Sun, W.; Wu, J.; Wang, Y.; Gamber, M.; Fan, L.; He, G., Do post-COVID-19 symptoms exist? A longitudinal study of COVID-19 sequelae in Wenzhou, China. *Ann Med Psychol (Paris)* **2021,** *179* (9), 818–821.
231. Shang, L.; Wang, L.; Zhou, F.; Li, J.; Liu, Y.; Yang, S., Long-term effects of obesity on COVID-19 patients discharged from hospital. *Immun Inflamm Dis* **2021,** *9* (4), 1678–1685.
232. Boscolo-Rizzo, P.; Menegaldo, A.; Fabbris, C.; Spinato, G.; Borsetto, D.; Vaira, L. A.; Calvanese, L.; Pettorelli, A.; Sonego, M.; Frezza, D.; Bertolin, A.; Cestaro, W.; Rigoli, R.; D'Alessandro, A.; Tirelli, G.; Da Mosto, M. C.; Menini, A.; Polesel, J.; Hopkins, C., Six-Month Psychophysical Evaluation of Olfactory Dysfunction in Patients with COVID-19. *Chem Senses* **2021,** *46*.
233. Dankowski, R.; Sacharczuk, W.; Duszyńska, D.; Mikołajewska, W.; Szałek-Goralewska, A.; Łojko-Dankowska, A.; Szyszka, A.; Łojko, D., Depression and anxiety in patients recently recovered from coronavirus disease (COVID-19). *Neuropsychiatria i Neuropsychologia* **2021,** *16* (1–2), 11–16.
234. Shendy, W.; Ezzat, M. M.; D.A, E. L.; Elsherif, A. A., Prevalence of fatigue in patients post Covid-19. *European Journal of Molecular and Clinical Medicine* **2021,** *8(3)*, 1330–1340.
235. Logue, J. K.; Franko, N. M.; McCulloch, D. J.; McDonald, D.; Magedson, A.; Wolf, C. R.; Chu, H. Y., Sequelae in Adults at 6 Months After COVID-19 Infection. *JAMA Netw Open* **2021,** *4* (2), e210830.
236. Ferrucci, R.; Dini, M.; Groppo, E.; Rosci, C.; Reitano, M. R.; Bai, F.; Poletti, B.; Brugnera, A.; Silani, V.; D'Arminio Monforte, A.; Priori, A., Long-Lasting Cognitive Abnormalities after COVID-19. *Brain Sci* **2021,** *11* (2), 1–11.
237. Tomasoni, D.; Bai, F.; Castoldi, R.; Barbanotti, D.; Falcinella, C.; Mule, G.; Mondatore, D.; Tavelli, A.; Vegni, E.; Marchetti, G.; d'Arminio Monforte, A., Anxiety and depression symptoms after virological clearance of COVID-19: A cross-sectional study in Milan, Italy. *J Med Virol* **2021,** *93* (2), 1175–1179.
238. Nair, C.; Moni, M.; Edathadathil, F.; Appukuttan, A.; Prasanna, P.; Roshni, P.; Sathyapalan, D., *Assessment of Post-Covid Symptoms in Covid-19 Recovered Patients: A Prospective Cohort Study in a Tertiary Care Centre of South India*. 2021.
239. Vanichkachorn, G.; Newcomb, R.; Cowl, C. T.; Murad, M. H.; Breeher, L.; Miller, S.; Trenary, M.; Neveau, D.; Higgins, S., Post-COVID-19 Syndrome (Long Haul Syndrome): Description of a Multidisciplinary Clinic at Mayo Clinic and Characteristics of the Initial Patient Cohort. *Mayo Clin Proc* **2021,** *96* (7), 1782–1791.

 © 2025. The Author(s).
